# An atlas of the tissue and blood metagenome in cancer reveals novel links between bacteria, viruses and cancer

**DOI:** 10.1186/s40168-021-01039-4

**Published:** 2021-04-22

**Authors:** Sven Borchmann

**Affiliations:** 1 Department I of Internal Medicine, Center for Integrated Oncology Aachen Bonn Cologne Duesseldorf, University of Cologne, Cologne, Germany; 2 Cancer Center Cologne Essen – Partner Site Cologne, CIO Cologne, University of Cologne, Cologne, Germany; 3 German Hodgkin Study Group, Cologne, Germany

## Abstract

**Background:**

Host tissue infections by bacteria and viruses can cause cancer. Known viral carcinogenic mechanisms are disruption of the host genome via genomic integration and expression of oncogenic viral proteins. An important bacterial carcinogenic mechanism is chronic inflammation. Massively parallel sequencing now routinely generates datasets large enough to contain detectable traces of bacterial and viral nucleic acids of taxa that colonize the examined tissue or are integrated into the host genome. However, this hidden resource has not been comprehensively studied in large patient cohorts.

**Methods:**

In the present study, 3025 whole genome sequencing datasets and, where available, corresponding RNA-seq datasets are leveraged to gain insight into novel links between viruses, bacteria, and cancer. Datasets were obtained from multiple International Cancer Genome Consortium studies, with additional controls added from the 1000 genome project. A customized pipeline based on KRAKEN was developed and validated to identify bacterial and viral sequences in the datasets. Raw results were stringently filtered to reduce false positives and remove likely contaminants.

**Results:**

The resulting map confirms known links and expands current knowledge by identifying novel associations. Moreover, the detection of certain bacteria or viruses is associated with profound differences in patient and tumor phenotypes, such as patient age, tumor stage, survival, and somatic mutations in cancer genes or gene expression profiles.

**Conclusions:**

Overall, these results provide a detailed, unprecedented map of links between viruses, bacteria, and cancer that can serve as a reference for future studies and further experimental validation.

Video Abstract

**Supplementary Information:**

The online version contains supplementary material available at 10.1186/s40168-021-01039-4.

## Introduction

Bacterial [1, 2] and viral [3–5] infections have widely been recognized as causes of cancer. Examples of carcinogenic viruses are Human Papillomaviridae, causing head and neck [6, 7] as well as cervical cancer [3, 8, 9] or *Hepatitis B virus*, causing liver cancer [10, 11]. The main carcinogenic mechanisms for viral carcinogenesis are thought to be (1) viral integration into and disruption of the host genome and (2) expression of oncogenic viral proteins [12].

An important example of bacteria causing cancer is *Helicobacter pylori* which can cause adenocarcinoma of the stomach [13, 14]. The carcinogenic mechanism at play here is thought to be an entirely different one compared to carcinogenic viruses, namely sustained inflammation caused by a chronic, mostly subclinical infection [1]. For some links between infections and cancer, preliminary evidence has been presented, but the simultaneous presence of contradictory findings has led to widespread debate. An example of this is the finding that high levels of *Fusobacterium nucleatum* can be found throughout the cancerous tissue of colorectal cancer at much higher levels than in the tissue of benign adenomas or healthy colon mucosa [15–17]. Given the diversity of carcinogenic mechanisms [18], it is likely that other carcinogenic viruses and bacteria exist, although currently unknown.

Recent advances in massively parallel sequencing have made it possible to generate large amounts of data informing about the genome, transcriptome, and epigenome of a tissue [19]. Resulting datasets contain traces of non-host origin that are present either because of genomic integration or the presence of the virus or bacteria in the tissue itself. While some studies have already been performed with the goal of repurposing this data in order to reveal novel links between infections and cancer [4, 5, 20, 21], these resources have so far been underutilized.

With the above aim in mind, the present study leverages a large, high-quality collection of over 3000 whole genome sequencing datasets in order to gain insight into novel links between viruses, bacteria, and cancer.

## Results

### Samples

**Fig. 1:**
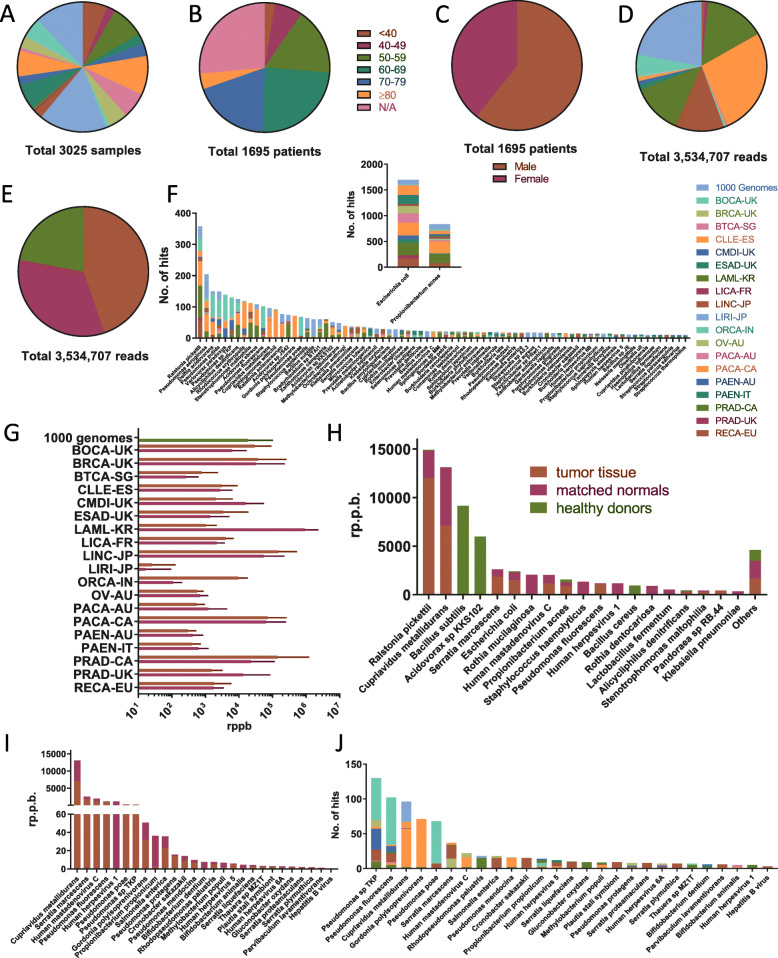
Overview of sample characteristics and identified taxa. **a** Sample distribution by project. **b** Sample distribution by age group. **c** Sample distribution by gender. **d** Distribution of read pairs matching any species-level taxa by project. **e** Distribution of read pairs matching any species-level taxa by sample type. **f** Distribution of total reads per sample, analyzed unmapped reads per sample, and non-*phiX174* taxon-mapped reads per sample (non-*phiX174* taxon-mapped reads per sample not shown for 365 samples as none were detected). **g** Species-level taxa detected in at least 10 samples color-coded by project. **h** Detection of *Escherichia coli* and *Propionibacterium acnes* color-coded by project. **i** Average read pairs per billion (RPPB) detected across all species-level taxa by project and sample type. Bars show mean of samples and error bars show standard error of mean. **j** Average RPPB detected by species-level taxa and sample type. Average RPPB detected for all filtered species-level taxa identified as likely tumor-linked by sample type. Filtered species-level taxa identified as likely tumor-linked color-coded by project. *, tumor tissue samples were all primary solid tumor biopsy material for all projects with the following exceptions: CLL-ES, where tumor tissue samples were blood-derived CLL cells; CMDI-UK, where 3 tumor tissue samples were bone-marrow-derived and 29 samples blood-derived; LAML-KR, where all 12 tumor tissue samples were bone-marrow-derived; PACA-AU, where 1 tumor tissue sample was cell-line-derived; and 96 solid tumor biopsy material and PRAD-UK, where 4 samples were cancerous lymph nodes, 1 sample was a metastatic lesion, and 28 samples were solid tumor biopsy material.**, matched normal samples were all blood-derived for CLL-ES, LINC-JP, ORCA-IN, PRAD-CA, and RECA-EU, all matched non-cancerous tissue derived for BTCA-SG, LICA-FR, and PAEN-IT, a mix between blood-derived and matched non-cancerous tissue for BOCA-UK (69 vs. 7), BRCA-UK (44 vs. 1), ESAD-UK (87 vs. 10), LIRI-JP (250 vs. 6), PACA-AU (3 vs. 94), PACA-CA (55 vs. 68), and PAEN-AU (5 vs. 44), a mix between blood-derived and buccal cell-derived for CMDI-UK (31 vs. 1), a mix between blood-derived and EBV immortalized cell-line-derived for OV-AU (59 vs. 14), and a mix between blood-derived and cancer-free lymph node derived for PRAD-UK (23 vs.10). For some samples, the source tissue was not specified (BRCA-UK (*n* = 1), LAML-KR (*n* = 1), and PACA-CA (*n* = 24). ***, all healthy donor samples are blood-derived

A total of 3025 whole genome sequencing datasets comprising 3.79 trillion reads were included in this study (Fig. 1a, Supplementary Table 1). These include 1330 whole genome sequencing datasets of tumor tissue samples across 14 different cancers from 19 International Cancer Genome Consortium (ICGC) [22] studies (Supplementary Table 1). Included patients were predominantly male (*n* = 1028, 60.6%) and elderly, with 47.3% of patients (*n* = 801) at least 60 years old (Fig. 1b,c). Two types of controls were used throughout this study. First, patient-matched normal (e.g., non-cancerous) tissue controls (*n* = 1330, mostly blood-derived or from tissue adjacent to the tumor, details in Supplementary Table 1) were utilized as controls. Only patients, for whom such a same-patient control was available were included in this study. Additionally, whole genome sequencing datasets of blood-derived DNA of 365 subjects from the 1000 genome project [23] were selected as a healthy control group substituting for the lack of negative sequencing controls to examine non-human DNA in the blood of healthy donors. Samples in the healthy control group were processed and sequenced at 5 different sequencing centers (86 at the BGI-Shenzhen, 86 at the Broad Institute, 11 at Illumina, 113 at the Sanger Institute, and 69 at Washington University in St Louis). Only subjects, in whom blood-derived DNA was directly subjected to whole genome sequencing, as opposed to DNA derived from immortalized lymphoblastoid cell lines (LCL) (subset analyzed, *n* = 102), were included in the healthy control cohort. Whole genome sequencing datasets derived from LCL DNA showed a markedly different species-level taxon distribution likely representing taxa present due to LCL culture and not present in the donor itself (Supplementary Figure 1A, Supplementary Data 1-2). For validation purposes and to assess differential gene expression in cancers linked to certain species-level taxa, all available, matching RNA-seq datasets were also analyzed (*n* = 324).

### Validation of pipeline

To perform taxonomic binning, a pipeline was built around Kraken [24], which at its core is based on the exact alignments of k-mers to their least common ancestor (LCA).

Kraken has been evaluated in two studies comparing metagenomic classifiers. In the first study [25], Kraken with its built-in filter performed well in species-level taxonomic binning, only being outperformed by few other tools, measured by F1 score, precision, recall, and area under the precision-recall curve. Importantly, it was only outperformed by tools that have a low recall if only very little sequence coverage is present for a taxon. These alternative tools are therefore not useful for this study. The same applies to combining different tools to arrive at a consensus binning. All tested combinations of other tools with Kraken have a very low recall rate at low coverage.

A low false positive rate is essential for this study and Kraken achieves this if its built-in filter is used. These performance characteristics were largely similar in another comparative study of metagenomic classifiers [26]; however, this, second study also found that the taxonomic binning by Kraken often contains false positive, very small bins. This can be mitigated by ignoring taxonomic bins that are very small, i.e., contain very few reads, which has been done in this study. Additionally, the filter stringency was increased. In one of the comparative studies of metagenomic classifiers, a filter threshold of 0.2 was used [25]. In the present study, a more stringent filter threshold of 0.5 was applied, aiming to further reduce false positives. Furthermore, the pipeline was validated twofold. In brief, it was confirmed that (i) the pipeline was able to identify already known bacterial and viral taxa in tissue-derived bacterial isolates and cell lines with known integration of viral DNA (Supplementary Data 2, Supplementary Figure 1B), and (ii) identified taxa in pairs of RNA-seq and WGS data of the same tumor tissue sample are correlated both in a combined dataset of all pairs and within each sample for which RNA-seq and WGS data was available (*n* = 324) (Supplementary Figure 1 C-D).

### A map of cancer-linked bacterial and viral taxa

A total of 3,534,707 read pairs matching bacterial, viral, or phage species-level taxa were detected across 19 studies in 3025 samples (Fig. 1d,e, Supplementary Table 1, Supplementary Data 4). Subsampling 10% of all read pairs did not alter the detected species-level taxa, and their relative composition compared to analyzing all non-human read pairs which was validated in a subset of patients (Supplementary Figure 1E-F, Supplementary Data 5). On average, 2.2 species-level taxa per sample were detected, although variation was high (Supplementary Data 6, Supplementary Figure 3 A-D). The mean number of total reads per sample was 1.25 × 10^9^ (1.16 × 10^7^ standard error of mean (s.e.m.)), the mean number of non-human, unmapped reads used for analysis per sample was 9.91 × 10^5^ (4.34 × 10^4^ s.e.m.), and the mean number of reads matching any taxon (excluding phiX174) was 2072 (291.8 s.e.m.) (Fig. 1f). A total of 218 species-level taxa could be identified in all examined samples (Fig. 1g, Supplementary Data 7). *Escherichia coli* and *Propionibacterium acnes* were the most detected species in all samples (Fig. 1h).

**Fig. 2:**
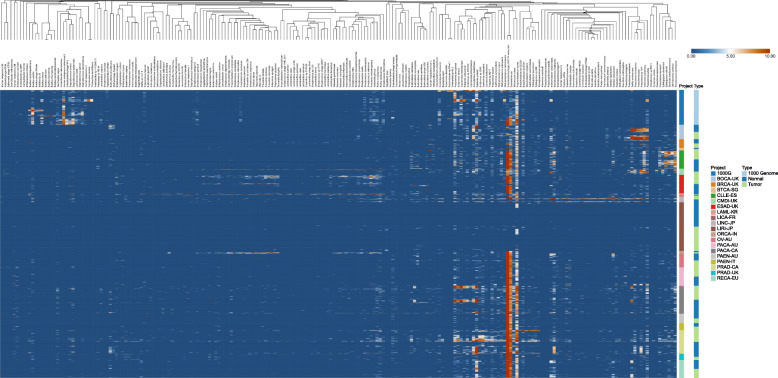
Heatmap of all taxa detected in all samples. Log2-transformed RPPB of all taxa in all samples (also including taxa that were filtered out subsequently as potential contaminants). Taxa were hierarchically clustered using Pearson correlation as a distance measure with average-linkage. Samples were hierarchically clustered within each project and type subgroup using Pearson correlation as a distance measure with average-linkage. Tumor tissue samples were all primary solid tumor biopsy material for all projects with the following exceptions: CLL-ES, where tumor tissue samples were blood-derived CLL cells; CMDI-UK, where 3 tumor tissue samples were bone-marrow-derived and 29 samples blood-derived; LAML-KR, where all 12 tumor tissue samples were bone-marrow-derived; PACA-AU, where 1 tumor tissue sample was cell-line-derived; and 96 solid tumor biopsy material and PRAD-UK, where 4 samples were cancerous lymph nodes, 1 sample was a metastatic lesion, and 28 samples were solid tumor biopsy material. Matched normal samples were all blood-derived for CLL-ES, LINC-JP, ORCA-IN, PRAD-CA, and RECA-EU, all matched non-cancerous tissue derived for BTCA-SG, LICA-FR, and PAEN-IT, a mix between blood-derived and matched non-cancerous tissue for BOCA-UK (69 vs. 7), BRCA-UK (44 vs. 1), ESAD-UK (87 vs. 10), LIRI-JP (250 vs. 6), PACA-AU (3 vs. 94), PACA-CA (55 vs. 68), and PAEN-AU (5 vs. 44), a mix between blood-derived and buccal cell-derived for CMDI-UK (31 vs. 1), a mix between blood-derived and EBV immortalized cell-line-derived for OV-AU (59 vs. 14), and a mix between blood-derived and cancer-free lymph node derived for PRAD-UK (23 vs.10). For some samples, the source tissue was not specified (BRCA-UK (*n* = 1), LAML-KR (*n* = 1), and PACA-CA (*n* = 24). All healthy donor samples are blood-derived

In order to control for differences in sequencing depth between samples, all raw read pair counts were normalized by dividing them by 1,000,000,000 total read pairs. This normalized count was defined as read pairs per billion (RPPB). The mean RPPB detected in healthy control samples from the 1000 genome cohort, matched normal samples, and tumor tissue samples were 18,112 (4238 s.e.m.), 20,003(4295 s.e.m.), and 28,282 (9081 s.e.m.), respectively, with large variation across samples and sequencing projects (Fig. 1i). Of note, particularly high RPPB were detected in matched normal, saliva-derived samples from acute myeloid leukemia (AML) patients, as would be expected from a non-sterile source such as saliva. *Ralstonia pickettii* was the species with the highest RPPB and almost absent in healthy donors, while being detected at higher levels in tumor tissue compared to matched normal samples (Fig. 1j). Except for *Bacillus subtilis*, *Acidovorax sp. KKS102*, and *Bacillus cereus*, all species with very high RPPB were detected predominantly in tumor tissue or matched normal samples. A clustered heatmap of the species-level taxa detected in all samples is provided in Fig. 2.

#### Filtering strategy

Next, filtering was performed to exclude taxa that were (i) frequently detected in the healthy control group, (ii) detected in only very few (< 5) tumor tissue or matched normal samples, (iii) phages, (iv) taxa that are commonly detected as part of the normal oral microbiome and were mainly detected in saliva, oral or esophageal cancer tissue samples (Supplementary Data 8), (v) taxa that have been previously described as sequencing contaminants (Supplementary Data 9), and (vi) taxa, for which the detected reads were unevenly distributed across the genome of the respective taxon (Supplementary Figure 4). After all these filtering steps (Supplementary Figure 5), 27 species-level taxa remained for further analysis (Supplementary Figure 6, Supplementary Data 10).

Among these, known tumor-linked taxa, such as *Hepatitis B virus* [10, 11] or *Salmonella enterica* [27, 28], were detected. Furthermore, taxa that have previously been implicated in carcinogenesis, although without enough evidence to support a carcinogenic role, such as *Pseudomonas* species [29, 30], and taxa that have never been implicated in carcinogenesis before, such as *Gordonia polyisoprenivorans*, were detected. Of note, most taxa in the final filtered species list were detected at much higher RPPB levels in tumor tissue compared to matched normal samples. However, lower RPPB of the respective species could still be detected in most matched normal samples. Across all taxa, tumor tissue samples and matched normal samples were highly correlated (Supplementary Figure 1G).

#### Pan-cancer analysis

In order to better understand the link between detected taxa and different cancers and their relation to each other, a heuristic approach combining the non-linear dimensionality reduction and visualization method *t*-distributed stochastic neighborhood embedding (t-SNE) with *k*-means clustering was used. To focus on taxa that potentially play a more direct role in carcinogenesis and considering that RPPB of detected taxa were highly correlated between tumor tissue and matched normal tissue with lower levels detected in matched normal tissue (Supplementary Figure 1G), only tumor tissues were included in the following analyses.

**Fig. 3:**
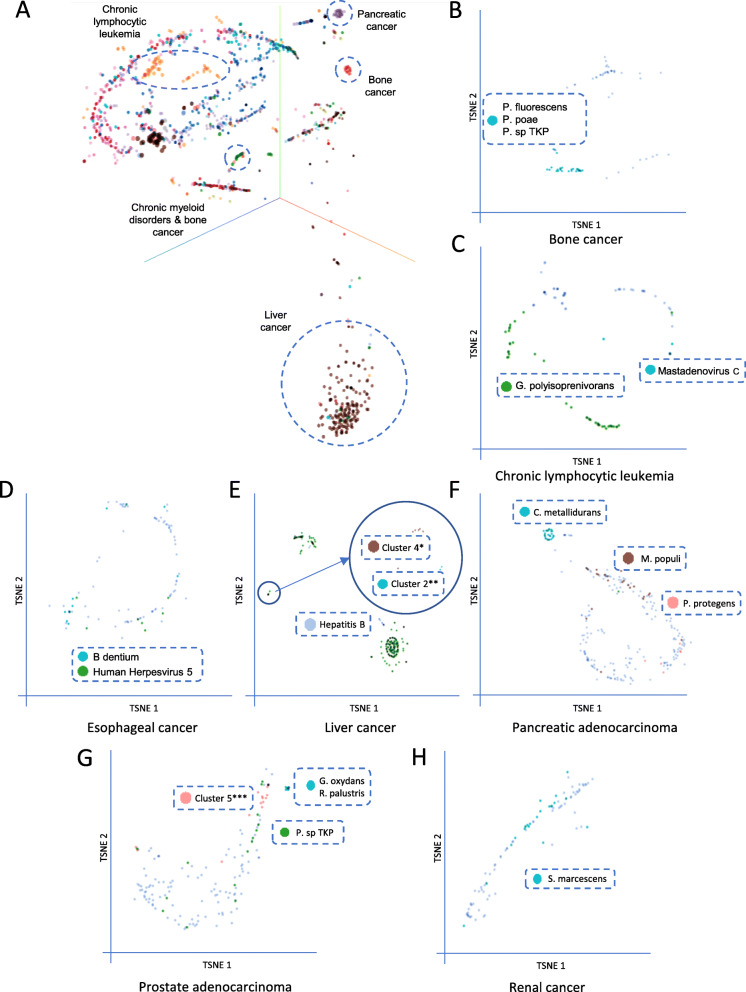
Patient clusters defined by detected taxa can be identified across all patients and in cancer-type subgroups. **a** t-SNE visualization of all tumor tissue samples color coded by project using the log_2_-transformed RPPB of all detected non-phage taxa as input variables. **b**–**h** t-SNE visualizations of single cancer subgroup tumor tissues (**b** bone cancer, **c** chronic lymphocytic leukemia, **d** esophageal cancer, **e** liver cancer, **f** pancreatic adenocarcinoma, **g** prostate adenocarcinoma, **h** renal cancer). Different colors indicate the *k*-means clusters of samples to indicate if *k*-means clustering and t-SNE visualization results in comparable sample groupings (Supplementary Figure 4). Log_2_-transformed RPPB of all detected non-phage taxa were used as input variables for t-SNE visualization. Dimension 1 and dimension 2 is shown on the *x*- and *y*-axis, respectively. Cluster 4*, *Pseudomonas sp.*, *Serratia sp.*, and *Salmonella enterica*; Cluster 2**, *Pseudomonas sp.*, *Serratia sp.*, *Salmonella enterica*, *Parvibaculum lavamentivorans*, and *Human Herpesvirus 5*; Cluster 5***, *Thauera sp. MZ1T*, *Cupriavidus metallidurans*, and *Pseudomonas mendocina*

Utilizing a pan-cancer approach, a combined dataset of all tumor tissue samples was visualized using t-SNE using the log_2_-transformed RPPB of all detected non-phage taxa (*n* = 204) as input variables. First, two distinct groups of patients with chronic lymphocytic leukemia (CLL) could be identified. One group (*n* = 24) with detection of *Gordonia polyisoprenivorans* and another group (*n* = 15) with detection of *Pseudomonas mendocina* in the tumor tissue. Second, one distinct group (*n* = 23) of pancreatic cancer patients with detection of *Cupriavidus metallidurans* was observed. Third, one group (*n* = 22) of bone cancer patients with detection of *Pseudomonas poae*, *Pseudomonas fluorescens*, and *Pseudomonas sp. TKP* could be distinguished. Fourth, a group of patients (*n* = 14) with bone cancer (*n* = 4) or chronic myeloid disorders (*n* = 10) with detection of *Pseudomonas sp. TKP* was identified (Fig. 3a). Of note, all taxa that defined clusters in this analysis where detected across cohorts with the same and cohorts with different cancers making contamination unlikely.

#### Cancer-specific analysis

In order to gain further insight into links between detected taxa and specific cancers, each cancer type (Supplementary Data 11) was also analyzed separately. Patient groupings with similar detected taxa were visualized using t-SNE with log_2_-transformed RPPB of all species in the final filtered taxon list as input variables (Supplementary Data 10). Additionally, *k*-means clustering of patients was performed, using the same input variables. To confirm associations and clusters, two statistical tests were performed. First, sample RPPBs of a taxon in an identified cluster were compared to RPPBs of all samples of the same cancer type not in that cluster by Mann-Whitney *U* test to confirm an abundance difference of samples in the respective cluster compared to samples not in that cluster (denoted *p*_cluster_). Second, sample RPPBs of a taxon in an identified cluster were compared to RPPBs of all samples in the healthy control cohort by Mann-Whitney *U* test to confirm the association of this taxon with a particular cancer (denoted *p*_control_).

Furthermore, each cluster was linked with age, survival, gender, number of somatic mutations in known cancer genes, or specific somatic mutations in one of those cancer genes. Aiming to increase the validity of findings, all identified links between certain taxa and cancers were evaluated for presence in multiple independent sample cohorts of the same cancer, wherever possible. Multiple sample cohorts where available for liver cancer (*n* = 3), prostate adenocarcinoma (*n* = 2), pancreatic adenocarcinoma (*n* = 2), and pancreatic endocrine neoplasms (*n* = 2).

#### Bone cancer

**Fig. 4:**
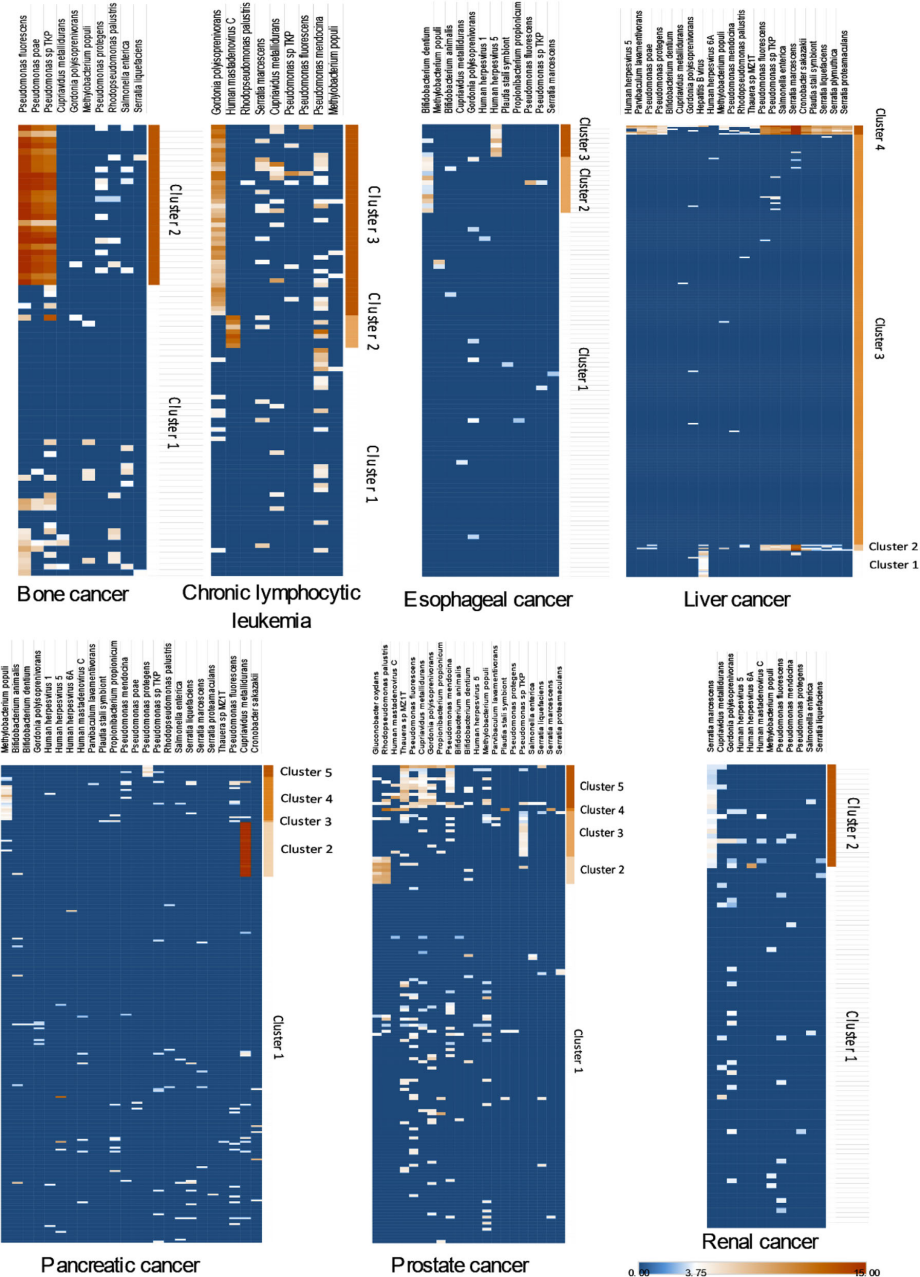
Heatmaps of tumor-linked taxa for all cancers with discernible clusters. **a–g** log_2_-transformed RPPB of all species-level taxa identified as likely tumor-linked and detected after filtering in all tumor-tissues of the indicated cancer. Results of *k*-means clustering of samples are shown

In bone cancer, this dual methodology revealed a cluster of patients with detection of *Pseudomonas fluorescens* (*p*_cluster_ = 3.2 × 10^−14^, *p*_control_ < 1 × 10^−15^), *Pseudomonas sp. TKP* (*p*_cluster_ = 1 < 10^−15^, *p*_control_ < 1 × 10^−15^), and *Pseudomonas poae* (*p*_cluster_ = 2.8 × 10^−13^, *p*_control_ < 1 × 10^−15^) (cluster 2), confirming the results of the pan-cancer analysis (Figs. 3 and 4b, a).

#### Chronic lymphocytic leukemia

**Fig. 5:**
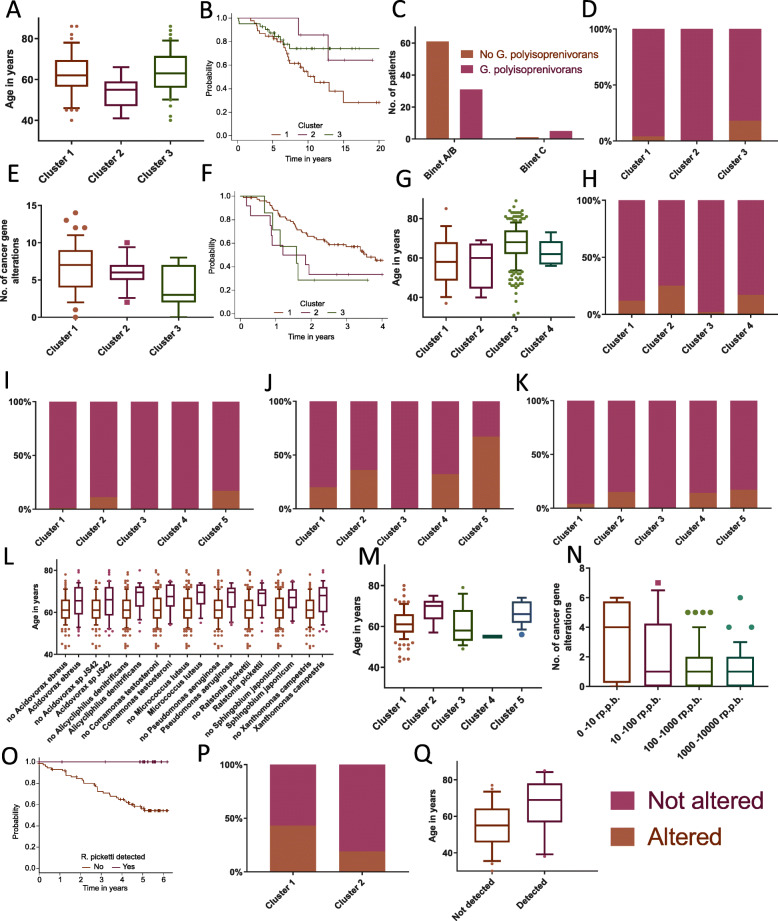
Patient clusters defined by detected taxa are phenotypically distinct. **a** CLL patient clusters (1: no specific taxon link, 2: *Human Mastadenovirus C*, 3: *Gordonia polyisoprenivorans*) and age (*p* = 0.0743). **b** Survival by cluster in CLL (*p*(1 vs. other) = 0.0246). **c** Binet stage by detection of *Gordonia polyisoprenivorans* (*p* = 0.0252). **d** TP53 mutation frequency by cluster in CLL (*p*(cluster 3 vs. other) = 0.0335). **e** Number of cancer consensus gene mutations by cluster (1: no specific taxon link, 2: *Bifidobacterium dentium*, 3: *Human Herpesvirus 5*) in esophageal cancer (*p* = 0.0858). **f** Kaplan-Meier survival curves for each cluster in esophageal cancer (*p*(1 vs. other) = 0.0409). **g** Liver cancer patient clusters (1: *hepatitis B virus*, 2: *Pseudomonas sp.*, *Serratia sp.* and *Salmonella enterica*, 3: no specific taxon link, 4: *Parvibaculum lavamentivorans* and *Human Herpesvirus 5* in addition to taxa from cluster 2) and age (*p* = 0.0015). **h** RNF21 mutation frequency by cluster in liver cancer (*p* = 0.0121). **i** KMT2C mutation frequency by cluster (1: no specific taxon link, 2: *Cupriavidus metallidurans*, 3: no specific taxon link, 4: *Methylobacterium populi*, 5: *Pseudomonas protegens*) in pancreatic cancer (*p* = 0.0308). **j** CDKN2A mutation frequency by cluster in pancreatic cancer (*p* = 0.0124). **k** RNF21 mutation frequency by cluster in pancreatic cancer (*p*(RNF21) = 0.0107). **l** Detection of indicated taxa and age in prostate cancer (*p*_adj_ between 0.0015 and 0.0420). **m** Prostate cancer patient clusters (1: no specific taxon link, 2: *Gluconobacter oxydans* and *Rhodopseudomonas palustris*, 3: *Pseudomonas sp. TKP*, 4: no specific taxon link, 5: *Thauera sp. MZ1T*, *Cupriavidus metallidurans* and *Pseudomonas mendocina*) and age (*p* = 0.0099). **n** Link between *Propionibacterium acne* RPPB and number of cancer consensus gene mutations in prostate cancer (*p*_adj_ = 0.0041). **o** Kaplan-Meier survival analysis of *Ralstonia pickettii* detection status in renal cancer (*p*_adj_ = 0.035). **p** PBRM1 mutation frequency by cluster (1: no specific taxon link, 2: *Serratia marcescens*) in renal cancer (*p* = 0.0723). **q** *Pseudomonas sp. TKP* detection and age in chronic myeloid dysplasia (*p*_adj_ = 0.039). For all: The midline of the boxplots shows the median, the box borders show upper and lower quartiles, the whiskers show 5th and 95th percentiles and the dots outliers. Only tumor tissues were included in this figure

In chronic lymphocytic leukemia, 2 taxon-linked clusters could be identified, one of patients with detection of *Human Mastadenovirus C* (*p*_cluster_ < 1 × 10^−15^, *p*_control_ < 1 × 10^−15^) (cluster 2) and one of patients with detection of *Gordonia polyisoprenivorans* (*p*_cluster_ = 1.5 × 10^−10^, *p*_control_ = 5 × 10^−15^) (cluster 3). Clusters could be identified by both methods, t-SNE and *k*-means (Fig. 3c, Supplementary Figure 3B). Of note, the clusters of patients linked to *Human Mastadenovirus C* (cluster 2) and *Gordonia polyisoprenivorans* (cluster 3) were mutually exclusive (*p* = 0.0124) (Figs. 3 and 4c, b). There was a tendency towards different ages at diagnosis between the clusters (*p* = 0.0743) (Fig. 5a), with patients in cluster 2 (*Human Mastadenovirus C*) being younger. Additionally, there was a tendency towards a difference in survival between the different clusters (*p* = 0.0745). Patients not in any taxon-linked cluster had worse survival than patients in cluster 2 (*Human Mastadenovirus C*) or 3 (*Gordonia polyisoprenivorans*) (*p* = 0.0246) (Fig. 5b). Patients linked to *Gordonia polyisoprenivorans* (cluster 3) were more likely to have Binet C stage disease (5/36 vs. 1/61, Binet C vs. not, *p* = 0.0252) (Fig. 5c). These patients were also more likely to have TP53 mutations (*p*(cluster 3 vs. other) = 0.0335) (Fig. 5d).

#### Esophageal cancer

In esophageal cancer patients, 2 taxon-linked clusters were identified. *k*-means clustering revealed one cluster of patients with detection of *Bifidobacterium dentium* (*p*_cluster_ = 1.7 × 10^−14^, *p*_control_ < 1 × 10^−15^) (cluster 2) and one cluster with detection of *Human Herpesvirus 5* (*p*_cluster_ = 7.8 × 10^−11^, *p*_control_ = 5 × 10^−15^) (cluster 3). However, these 2 clusters could not be differentiated by t-SNE (Figs. 3 and 4d, c). There was a tendency towards different numbers of somatic mutations in cancer genes between clusters (*p* = 0.0858), with patients in cluster 3 (*Human Herpesvirus 5*) having fewer mutations than other patients (*p* = 0.0392) (Fig. 5e). Additionally, there was a tendency towards a difference in survival between the different clusters (*p* = 0.1). Patients in cluster 2 (*Bifidobacterium dentium*) or 3 (*Human Herpesvirus 5*) had worse survival than patients not in any taxon-linked cluster (*p* = 0.040928). (Fig. 5f).

#### Liver cancer

Patients with liver cancer could be grouped into 3 taxon-linked clusters by both *k*-means clustering and t-SNE. One cluster was defined by detection of *Hepatitis B virus* (*p*_cluster_ < 1 × 10^−15^, *p*_control_ < 1 × 10^−15^) (cluster 1). A second cluster was defined by detection of mainly *Pseudomonas* (*p*_cluster_ between 1.6 × 10^−6^ and 4.5 × 10^−6^, *p*_control_ 1.3 × 10^−9^ for all) and *Serratia species* (*p*_cluster_ between 2.8 × 10^−6^ and 1.1 × 10^−4^, *p*_control_ between 1.3 × 10^−9^ and 4.8 × 10^−7^) and *Salmonella enterica* (*p*_cluster_ = 7.0 × 10^−7^, *p*_control_ = 1.3 × 10^−9^) (cluster 2). A third cluster was defined by additional detection of *Parvibaculum lavamentivorans* (*p*_cluster_ = 1.2 × 10^−12^, *p*_control_ = 2.9 × 10^−13^) and *Human Herpesvirus 5* (*p*_cluster_ = 5.0 × 10^−8^, *p*_control_ = 1.9 × 10^−8^) as well as two additional *Pseudomonas species*, *Pseudomonas poae* (*p*_cluster_ = 1.2 × 10^−12^, *p*_control_ = 2.9 × 10^−13^), and *Pseudomonas protegens* (*p*_cluster_ = 1.2 × 10^−12^, *p*_control_ = 2.9 × 10^−13^) (cluster 4), in addition to those detected in the previous cluster (Figs. 3 and 4e, d). *Hepatitis B virus* and *Serratia species* were detected in 2 independent cohorts, while abovementioned *Pseudomonas species* were detected in all 3 independent cohorts, making contamination unlikely. There was a tendency towards different ages at diagnosis between the clusters (*p* = 0.0015) with patients in cluster 1 (*Hepatitis B*), cluster 2 (*Pseudomonas* and *Serratia* sp.), and cluster 4 (*Pseudomonas* sp., *Serratia* sp., *Parvibaculum lavamentivorans*, and *Human Herpesvirus 5*) being younger than patients not in any taxon-linked cluster (*p* = 0.0001) (Fig. 5g). In a similar pattern, these patients had a higher frequency of mutations in RNF21 (*p* = 0.0121) (Fig. 5h).

#### Pancreatic adenocarcinoma

In patients with pancreatic adenocarcinoma, 3 taxon-linked clusters could be identified using *k*-means clustering. One cluster was defined by detection of *Cupriavidus metallidurans* (*p*_cluster_ < 1 × 10^−15^, *p*_control_ < 1 × 10^−15^) (cluster 2), one by detection of *Methylobacterium populi* (*p*_cluster_ < 1 × 10^−15^, *p*_control_ < 1 × 10^−15^) (cluster 4), and a last one by detection of *Pseudomonas protegens* (*p*_cluster_ = 3.6 × 10^−12^, *p*_control_ = 2.9 × 10^−13^) (cluster 5). Only the cluster of patients linked to *Cupriavidus metallidurans* (cluster 2) could also be identified using t-SNE (Figs. 3 and 4f, e). All 3 taxon-species associations could be detected in both independent sample cohorts that were included, making contamination unlikely. Differences in the frequency of mutations between patients in different clusters were observed in KMT2C, CDKN2A, and RNF21. Patients in cluster 2 (*Cupriavidus metallidurans*), 4 (*Methylobacterium populi*), or 5 (*Pseudomonas protegens*) had a higher frequency of mutations in these genes compared to the majority of patients not in any taxon-linked cluster (*n* = 187) (*p* (KMT2C) = 0.0308, *p* (CDKN2A) = 0.0124, *p* (RNF21) = 0.0107) (Fig. 5i–k).

#### Prostate cancer

In patients with prostate cancer, 3 taxon-linked clusters emerged using *k*-means clustering, one defined by detection of *Gluconobacter oxydans* (*p*_cluster_ = 6.6 × 10^−13^, *p*_control_ < 1 × 10^−15^) and *Rhodopseudomonas palustris* (*p*_cluster_ = 8 × 10^-15^, *p*_control_ < 1 × 10^−15^) (cluster 2), one by detection of *Pseudomonas sp. TKP* (*p*_cluster_ < 1 × 10^−15^, *p*_control_ < 1 × 10^-15^) (cluster 3) and a last one by detection of *Thauera sp. MZ1T* (*p*_cluster_ = 1.3 × 10^−11^, *p*_control_ < 1 × 10^−15^), *Cupriavidus metallidurans* (*p*_cluster_ < 1 × 10^−15^, *p*_control_ < 1 × 10^−15^), and *Pseudomonas mendocina* (*p*_cluster_ = 1.8 × 10^-8^, *p*_control_ < 1 × 10^-15^) (cluster 5). Out of these, the cluster defined by *Gluconobacter oxydans* and *Rhodopseudomonas palustris* (cluster 2) and the one defined by *Thauera sp. MZ1T*, *Cupriavidus metallidurans*, and *Pseudomonas mendocina* (cluster 5) could be confirmed using t-SNE (Figs. 3 and 4g, f). *Cupriavidus metallidurans*, *Rhodopseudomonas palustris*, and *Pseudomonas species* could be detected in both independent sample cohorts that were included. However, *Gluconobacter oxydans* could only be detected in one cohort. There were age differences at diagnosis observable between the clusters (*p* = 0.0099) with patients in clusters 2 (*Gluconobacter oxydans*) and 5 (*Rhodopseudomonas palustris*) being older than the other patients (*p* = 0.0005) (Fig. 5m).

#### Renal cancer

In patients with renal cancer, a cluster of patients linked to *Serratia marcescens* (*p*_cluster_ < 1 × 10^−15^, *p*_control_ < 1 × 10^−15^) (cluster 2) was identified using *k*-means clustering, although t-SNE did not separate this group of patients (Figs. 3 and 4h, g). There was a tendency towards a lower frequency of PBRM1 mutations in patients in cluster 2 (*Serratia marcescens*) (*p* = 0.0723) (Fig. 5p).

#### Other cancers

In patients with pancreatic endocrine neoplasms, ovarian cancer, chronic myeloid disorders, and breast cancer, no discernable taxon-linked clusters could be identified (Supplementary Figure 7A-D).

### Unbiased linkage analysis between bacterial and viral taxa and patient or cancer phenotypes

In addition to linking the above identified clusters with patient or cancer phenotypes, an unbiased analysis of links between the detection of a species-level taxa and patient or cancer phenotypes, such as age, survival, gender, number of somatic mutations in known cancer genes, and specific somatic mutations in one of those cancer genes, was performed utilizing both a pan-cancer approach and by analyzing each cancer type separately. In this analysis, all non-phage taxa detected (*n* = 204) were included and multiple testing correction was performed.

When analyzing cancer types separately, several links were identified. A group of bacterial taxa was linked to older patients in prostate cancer (*p*_adj_ between 0.0015 and 0.0420) (Fig. 5l). In chronic myeloid dysplasia, detection of *Pseudomonas sp. TKP* was also linked to older age (*p*_adj_ = 0.039) (Fig. 5q).

In the cancer-specific analysis, detection of *Ralstonia pickettii* was linked to improved survival in renal cancer, in fact no patients died (*p*_adj_ = 0.035) (Fig. 5o).

In prostate cancer, detection of and increasing *Propionibacterium acne* RPPB were linked to a decreasing number of cancer gene mutations (*p*_adj_ = 0.0041) (Fig. 5n).

### Somatic lateral gene transfer

The integration of viral nucleic acids into the human host genome is well recognized as a carcinogenic process. High-level evidence exists for the integration of *Hepatitis B* [10, 11], *Human Papillomavirus* [8, 9], and *Epstein–Barr virus* (*Human Herpesvirus 4*) [31, 32], which are causally linked to hepatocellular carcinoma, cervical cancer, and lymphoma, respectively. Additionally, some evidence for somatic lateral gene transfer from bacteria to cancerous tissue has already been presented [20] and subsequently controversially discussed.

Aiming to find evidence for bacterial or viral DNA integration into the human host genome in this dataset, a pipeline for this purpose was developed. In brief, read pairs in which one read mapped to the human genome and one read to one of the taxa in the final filtered taxon list (*n* = 27) were counted and then compared to the number of read pairs in which both reads mapped to the respective taxa. The number of divergently mapping read pairs divided by the number of complete pairs mapping that respective taxa was used as a measure for genomic integration and lateral gene transfer. This was done for the tumor tissue datasets and the matched normal datasets including all patients (*n* = 79) for which the pipeline revealed at least 1000 RPPB matching one of the taxa in the final filtered taxon list (Supplementary Data 12). The highest rate of integration was observed for *Hepatitis B* in tumor tissue (9.62%) (Supplementary Figure 8A, Supplementary Data 13). Across all analyzed taxa, putative integrations were more common in matched normal samples than in tumor tissue samples (*p* = 0.0009, Wilcoxon paired signed rank test) (Supplementary Figure 8B, Supplementary Data 13-14) and for viral taxa compared to bacterial taxa (*p* = 0.0001, Mann-Whitney *U* test) (Supplementary Figure 8C, Supplementary Data 13-14). In conclusion, there was no evidence for a general phenomenon of lateral gene transfer for any species in the final filtered taxon list, with the exception of *Hepatitis B*, for which integration into the cancer genome has been widely described [10, 11].

### Differential gene expression analysis

**Fig. 6:**
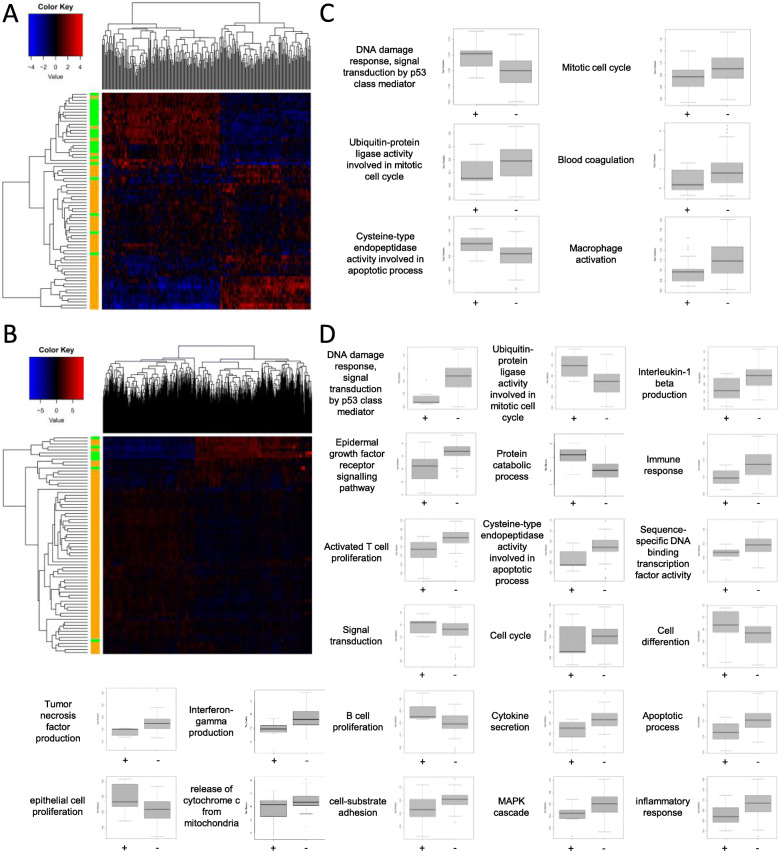
Differential gene expression and pathway analysis of chronic lymphocytic leukemia patients. **a** Heatmap of all differentially expressed genes (*q* < 0.05) in tumor tissue samples of chronic lymphocytic leukemia (CLL) patients with (green) and without (orange) detection of *Gordonia polyisoprenivorans*. Dendrograms show clustering with complete linkage and Euclidian distance measure. **b** Heatmap of all differentially expressed genes (*q* < 0.05) in tumor tissue samples of CLL patients with (green) and without (orange) detection of Human Mastadenovirus C. **c** Boxplots of the distribution of activating versus inhibitory gene *t*-test statistics for the indicated pathway is shown for tumor tissue samples of CLL patients with (+, *n* = 23) and without (−, *n* = 49) detection of *Gordonia polyisoprenivorans*. The midline of the boxplot shows the median, the box borders show upper and lower quartiles and the whiskers the maximum and minimum test statistic. **d** Boxplots of the distribution of activating versus inhibitory gene *t*-test statistics for the indicated pathway is shown for tumor tissue samples of CLL patients with (+, *n* = 7) and without (−, *n* = 65) detection of *Human Mastadenovirus C*. The midline of the boxplot shows the median, the box borders show upper and lower quartiles, and the whiskers the maximum and minimum test statistic. Dendrograms show clustering with complete linkage and Euclidian distance measure

Differential gene expression analysis was performed for all taxa and cancer combinations with available tumor tissue RNA-seq data, in which at least 5 patients had upwards of 100 RPPB matching the respective taxon (Supplementary Data 15). Differentially expressed genes (*q* < 0.05) were identified for chronic lymphocytic leukemia patients with or without detection of *Gordonia polyisoprenivorans* (258 genes) (Fig. 6a, Supplementary Data 16), *Human Mastadenovirus C* (1725 genes) (Fig. 6b, Supplementary Data 17), and *Pseudomonas aeruginosa* (50 genes, Supplementary Data 18), respectively. Furthermore, differentially expressed genes were identified for ovarian cancer patients with or without the detection of *Escherichia coli* (22 genes) (Supplementary Data 19) and pancreatic adenocarcinoma patients with or without detection of *Propionibacterium acnes* (3 genes) (Supplementary Data 20).

Next, differential gene expression data was used to perform bidirectional functional enrichment in order to identify pathways that are altered in patients with or without detection of the respective taxon. Only the two patient groups with a significant number of differentially expressed genes, chronic lymphocytic leukemia with or without detection of *Gordonia polyisoprenivorans* (Supplementary Data 21) or *Human Mastadenovirus C* (Supplementary Data 22) had enriched pathways.

Overall, patients with detection of *Gordonia polyisoprenivorans* exhibited a gene expression pattern indicative of a decreased level of mitotic cell cycling, an increased DNA damage response, reduced blood coagulation, and reduced macrophage activation (Fig. 6c).

Contrary to that, patients with detection of *Human Mastadenovirus C* exhibited a gene expression pattern indicative of an increase in B cell proliferation, a reduction of tumor necrosis factor and interleukin beta 1 production, a reduction of activated T cell proliferation, and a decrease in cytokine secretion. In addition, the altered tumor tissue gene expression pattern of patients with detection of *Human Mastadenovirus C* indicated a markedly reduced DNA damage response (Fig. 6d).

## Discussion

The aim of this this study was to leverage a large, high-quality dataset of over 3000 samples to reveal novel links between viral and bacterial taxa and cancer. A total of 218 species-level taxa could be identified in tumor tissue, matched normal and healthy donor samples. Out of these, following extensive filtering, 27 taxa were likely cancer-linked. While studies of the viral metagenome of cancer tissues and patients have been performed using datasets from cancer genomics studies [4, 5, 21], similar large studies examining the bacterial metagenome are lacking.

Studies examining the viral metagenome of cancer tissues mainly identified known links of *Human Papillomavirus* to cervical and head and neck cancer, of *Hepatitis B* to liver cancer, and of *Human Herpesvirus 5* to a variety of cancers while the detection of *Human Mastadenovirus C* was controversial [4, 5, 21].

Smaller studies examining bacteria-tumor links in pan-cancer datasets have identified *Escherichia coli*, *Propionibacterium acne*, and *Ralstonia pickettii* in multiple cancers, while more specifically finding *Acinetobacter sp.* in AML and *Pseudomonas sp.* in both AML and adenocarcinoma of the stomach [20, 33]. Studying cancer-specific datasets, *Salmonella enterica*, *Ralstonia pickettii*, *Escherichia coli*, and *Pseudomonas sp.* were detected in breast cancer and adjacent tissue [34], while *Escherichia sp.*, *Propionibacterium sp.*, *Acinetobacter sp.*, and *Pseudomonas sp.* were frequently detected in prostate cancer [30]. Confirming the findings of the present study, these smaller studies found similar bacterial taxa, especially *Ralstonia pickettii*, *Escherichia coli*, *Propionibacterium acne*, *Salmonella enterica*, and *Pseudomonas sp.* Interestingly, the present study identified a number of taxa that have not been previously identified in cancer tissue or matched normal samples of these patients, among them *Cupriavidus metallidurans*, *Gordonia polyisoprenivorans*, *Serratia sp.*, and *Bifidobacterium sp..* Reasons for this are likely (1) the much higher number and diversity of patients and samples included, (2) the larger amounts of data examined per sample due to having higher-coverage WGS datasets available for all patients compared to RNA-seq or whole-exome sequencing (WXS) datasets in previous studies, and (3) the optimized and extensively validated bioinformatics approach used here. Of note, the filtering strategy used in this study to exclude taxa likely resulting from contamination has also eliminated bacterial taxa that have previously been linked to cancer such as *Escherichia coli* and *Propionibacterium acne*, mainly due to the frequent detection of these taxa in healthy donor samples from the 1000 genome cohort. Stringent filtering for species-level taxa that were detected with at least 100-fold higher RPPB in tumor tissue or matched normal tissue compared to healthy donor samples excluded these species. Despite that, *Propionibacterium acne* had about 1.4× and 3.1× higher RPPB for matched normal and tumor tissue, respectively, compared to healthy donors. These values were even higher in the case of *Escherichia coli*, namely 7.1× and 13.4× for matched normal and tumor tissue, respectively. Thus, it is well possible that tumor-linked taxa were eliminated due to the stringent filtering utilized. Therefore, some analyses in this study, such as the unbiased linkage analysis between detected taxa and phenotypes, were performed using the unfiltered dataset and all raw data is provided along with this manuscript for further analysis with different filtering approaches.

Examining specific cancer-pathogen links, a subgroup of bone cancer patients with detection of *Pseudomonas sp.* in the tumor tissue was identified. Consistent links between viral or bacterial taxa and bone cancer have not been described before, apart from some evidence linking *simian virus 40 (SV40)* infection to bone cancer [35]. Interestingly, *Pseudomonas sp.* are frequently implied in difficult to treat cases of osteomyelitis. Thus, one might speculate that chronic, subclinical infection exists and could be carcinogenic.

For CLL patients, two links were identified, one to *Gordonia polyisoprenivorans* and one to *Human Mastadenovirus C. Gordonia polyisoprenivorans* has not been linked to cancer before. Interestingly, the bacterium has been identified as a rare cause of bacteremia, so far exclusively in patients with hematological cancers [36–38]. Hematological cancers and their treatment are often associated with profound immunosuppression, allowing for infections with unusual environmental pathogens. It is conceivable that these patients had a latent infection with *Gordonia polyisoprenivorans* which exacerbated into bacteremia and sepsis upon treatment-induced immunosuppression. To date, *Human Mastadenovirus C* has not been clearly linked to cancer, although recent reports have found it frequently detected in various cancer tissues, with one treating it as contamination [4]. Further indirect evidence for a role of both *Gordonia polyisoprenivorans* and *Human Mastadenovirus C* in CLL carcinogenesis is provided by (1) the observed age difference—patients with detection of *Human Mastadenovirus C* were markedly younger, (2) the mutual exclusivity of detection of *Gordonia polyisoprenivorans* and *Human Mastadenovirus C*, and (3) the fact that survival was different—patients with detection of either taxa had improved outcome. This was despite the higher likelihood of patients linked to *Gordonia polyisoprenivorans* having Binet C stage and patients linked to *Gordonia polyisoprenivorans* having a higher likelihood of having TP53 mutations, which are prognostically disadvantageous in CLL [39]. Strikingly, marked differences in host cancer tissue gene expression were observed for patients in which one of the taxa was detected with differences between cases with detection of *Gordonia polyisoprenivorans* and *Mastadenovirus C*. The observed tumor tissue gene expression pattern for patients linked to *Gordonia polyisoprenivorans*, especially an increased DNA damage response and reduced mitotic cell cycling, could explain the improved survival of these patients. The tumor tissue gene expression pattern of patients linked to *Human Mastadenovirus C* pointed towards reduced immune activity, especially reduced T cell function and a decrease in cytokine production and secretion, providing a potential explanation for the observed high detection frequency of *Human Mastadenovirus C* DNA in line with an uncontrolled infection due to a diminished immune response. Detection of *Mastadenovirus C* was linked to a decreased DNA damage response, which has been described as an important pathomechanism of CLL [40]. In addition to CLL patients, both *Gordonia polyisoprenivorans* and *Human Mastadenovirus C* were also detected frequently in other cancers, sometimes at high levels, while never being detected in healthy donors.

Two taxon-tumor links in esophageal cancer were identified, one to *Bifidobacterium dentium* and one to *Human Herpesvirus 5*. Interestingly, patients with detection of *Human Herpesvirus 5* had less somatic mutations in cancer consensus genes than the other patients, pointing to a possibly different carcinogenic mechanism, where the accumulation of multiple somatic mutations is not stringently needed for malignant transformation. Furthermore, patients not in any taxon-linked cluster had better survival. While the link of *Human Herpesvirus 5* to esophageal cancer is a novel finding, *Human Herpesvirus 5* has frequently been detected in adjacent adenocarcinoma of the stomach [5]. Of note, overt *Human Herpesvirus 5* esophagitis can occur in immunocompromised hosts [41], which could be indicative of a latent infection of the esophagus by *Human Herpesvirus 5* in some hosts.

In patients with liver cancer, one subgroup of patients was defined by detection of *Hepatitis B*, a known cause of liver cancer [10, 11]. Interestingly, two other subgroups could be identified. *Pseudomonas sp.* and *Serratia sp.* were detected in both groups, with additional detection of *Parvibaculum lavamentivorans* and *Human Herpesvirus 5* in one group. There is some evidence that *Human Herpesvirus 5* might play a role in the carcinogenesis of liver cancer, among them detection of *Human Herpesvirus 5* DNA in tumor tissue and an increased seroprevalence in liver cancer patients [42] as well as frequent hepatitis in *Human Herpesvirus 5* infection underscoring hepatotropism of *Human Herpesvirus 5* [43]. The other identified taxa have not yet been implied in liver cancer carcinogenesis. Interestingly, hepatocellular carcinoma at younger age has been linked to chronic *Hepatitis B* infection [44]. Similarly, younger age of onset was also observed in liver cancers linked to the other taxa in this study.

Three bacteria-tumor links were identified in pancreatic adenocarcinoma, one with *Pseudomonas protegens*, one with *Methylobacterium populi* and one with *Cupriavidus metallidurans*. While *Pseudomonas sp.* have been shown to be a contributor to the pancreatic adenocarcinoma tissue microbiome [29], *Methylobacterium populi* and *Cupriavidus metallidurans* have not been detected in pancreatic cancer tissue. In fact, both taxa have not been discovered in human hosts but in environmental samples and are thus not considered part of the human microbiome, making it possible that they are contaminants not truly present in the tumor tissue samples analyzed. On the other hand, few infections of humans by these taxa have been described [45, 46] and, of note, the first published report of a *Cupriavidus metallidurans* infection was a case of septicemia in a patient with a pancreatic tumor [46].

In patients with prostate cancer, the most interesting findings were age differences between patient clusters defined by the detection of different taxa and a negative correlation of *Propionibacterium acne* detection and number of mutations in cancer consensus genes. Patients with detection of *Gluconobacter oxydans* and *Rhodopseudomonas palustris* as well as patients with detection of *Thauera sp. MZ1T*, *Cupriavidus metallidurans*, and *Pseudomonas mendocina* were markedly older than the other patients. *Gluconobacter sp.*, *Rhodopseudomonas sp.*, *Cupriavidus sp.*, and *Pseudomonas sp.* have previously been identified in prostate cancer and normal prostate tissue [30], but their precise role in prostate disease is entirely unclear. *Propionibacterium acne* has been implied as a potential carcinogenic bacterium in prostate cancer [47, 48], possibly by creating a chronic inflammatory microenvironment [49]. In this study, the *Propionibacterium acne* detection frequency correlated negatively with the number of somatic mutations in cancer consensus genes. This could point to an alternative driver of carcinogenesis by chronic inflammation in the absence of accumulation of many mutations in cancer driver genes.

In renal cancer, a link to *Serratia marcescens* was identified in a subgroup of patients. While *Serratia marcescens* has been described as a frequent cause of urinary tract infections, especially in immunocompromised hosts in a nosocomial setting [50], it has so far not been implicated in cancer. Interestingly, survival of patients with detection of *Ralstonia pickettii* in their tumor tissue was markedly improved*. Ralstonia pickettii* is a bacterium that has been filtered out in this study because of frequent detection in healthy donor samples. It could be speculated that, while not causing any overt infection, low-level presence of *Ralstonia pickettii* in the human host is common and improves immunogenicity of renal cancer, thus, improving outcome. It has been shown that alterations of local and systemic immunity by the host microbiome influence the anticancer immune response [51], which might be highly relevant for a naturally immunogenic tumor, such as renal cancer [52].

The intriguing observation of increased somatic bacteria-human lateral gene transfer by Riley et al. [20] could not be made in this study. The only taxa for which more integration into the host genome was observed in tumor tissue compared to matched normal was *Hepatitis B*, for which integration into the genome of cancerous cells has been well recognized [53]. Additional integration of gut microbiome data would enhance this manuscript. The gut microbiome has recently emerged as being highly relevant to carcinogenesis, especially of cancers exposed to it, such as colorectal cancer [54]. It has also emerged that the gut microbiome can modulate cancer treatment efficacy, particularly of immunotherapy [55]. However, gut microbiome data was not available for the patients included in this study.

It is important to note, that this study is an explorative analysis of potential novel relationships between cancers, viruses, and bacteria and only experimental validation can really prove the postulated links of bacterial and viral taxa to certain cancers. Nevertheless, every attempt has been made to reduce false positives, by carefully choosing a pipeline based on available comparison studies [25, 26], applying stringent filtering, removing low complexity sequence, and removing taxonomic bins with only very few hits. Looking at genetic variation on the strain level between different patients could further validate the findings and rule out contamination [56]. However, this is not possible, as methods to do so all require a minimum coverage of the taxon in question. Due to the nature of this study—examining low-level presence of viruses or bacteria in cancer tissues—sufficient coverage is not available. Further validation could come from validating some of the findings on long-read sequencing platforms [57]. However, this study reused datasets already available and had no access to the original samples; therefore, such a further validation is not possible.

## Conclusions

In conclusion, the present study provides an unprecedented atlas of links of both bacterial and viral taxa to cancer. In addition to confirming known or recently postulated links, several novel associations between, bacteria, viruses, and cancer were identified across multiple cancer entities, laying the groundwork for further studies and experimental validation.

## Methods

### Data sources and data availability

Mapped sequencing data for the included ICGC studies was obtained via the ICGC DCC [22, 58] and downloaded using customized scripts. The use of controlled access ICGC data for this project was approved by the ICGC data access compliance office. Mapped sequencing data for the 1000 genome healthy control samples was obtained from the 1000 genome FTP server [59] using customized scripts. Sequencing data used for validation was obtained from the European Nucleotide Archive (ENA) [60]. Each sample is clearly identified by the identifiers in Supplementary Data 4. These identifiers can be used to obtain further sample and donor information from the ICGC data portal (https://dcc.icgc.org/). While basic data is available without approval, downloads of raw sequencing data via the ICGC data portal have to be requested from the ICGC data access compliance office. Raw read counts and analysis data are available in full and included in Supplementary Data 4.

### Computing environment

Data analysis was performed using a HP Z4 workstation in a Unix environment either using software as mentioned throughout the “Methods” section or customized scripts. Some analyses were performed employing the Galaxy platform [61].

### Pipeline for taxonomic classification

First, unmapped (non-human) read pairs were extracted from a random 10% subsample of each sample’s downloaded sequencing data using Samtools (version 1.7) [62]. Subsampling 10% did not alter the detected species-level taxa and their relative composition (Supplementary Figure 1E-F, Supplementary Data 5). Bam files were sorted by query name with Picard Tools (version 2.7.1.1) [63] and converted to FastQ files using Bedtools (version 2.26.0.0) [64]. Subsequently, Trimmomatic (version 0.36.3) [65] was used to trim reads with sliding window trimming with an average base quality of 20 across 4 bases as cutoff and dropping resulting reads with a residual length < 50. Read pairs, in which one read was dropped according to these rules, were dropped altogether. Remaining read pairs were joined with FASTQ joiner (version 2.0.1) [66] and converted to FASTA files using the built-in function FASTQ to FASTA (version 1.0.0) from Galaxy [61]. Next, VSearch (version 1.9.7.0) [67] was used to mask repetitive sequences by replacing them with Ns using standard settings. These masked and joined read pairs were fed into Kraken (version 1.1.1) [24] using a database of all bacterial and viral genomes in Refseq (release 85). The output of each run was filtered with Kraken (version 1.1.1) [24] setting a confidence threshold of 0.5. A report combining the output of all samples and runs was generated using Kraken (version 1.1.1) [24]. The output was then arranged using customized scripts in R (beginning with version R 3.3.2. and subsequently updated) [68] to generate the raw metagenome output of each sample. Next, the raw metagenome output of each sample was filtered by only including species-level taxa and excluding all species-level taxa that were supported by less than 10 read pairs across all samples using R (beginning with version R 3.3.2. and subsequently updated) [68]. Read pairs assigned to Enterobacteria phage phiX174, which is ubiquitously used as a spike-in control in next-generation sequencing were omitted from all counts and analyses as an intended contaminant, except for Fig. 2, which aims to visualize the full, raw dataset.

To correct for the variation of sequencing depth across samples, matched read pairs per billion read pairs raw sequence (RPPB) were calculated for each sample and each taxon.

RPPB was calculated using the following formula:

$$ {10}^9\times \frac{\mathrm{read}\ \mathrm{pairs}\ \mathrm{of}\ \mathrm{a}\ \mathrm{sample}\ \mathrm{assigned}\ \mathrm{to}\ \mathrm{a}\ \mathrm{given}\ \mathrm{taxon}}{\mathrm{total}\ \mathrm{available}\ \mathrm{read}\ \mathrm{pairs}\ \mathrm{for}\ \mathrm{a}\ \mathrm{given}\ \mathrm{sample}} $$

A taxon was heuristically considered detected, when a respective sample had at least 100 RPPB assigned to that taxon.

### Filtering strategy

First, all taxa that were also highly prevalent in the healthy control group were excluded from further analysis. In detail, a taxon was required to have a mean RPPB across either the tumor tissue samples or the matched normal samples compared to the healthy control samples of at least 100-fold higher, to be included. This cutoff excluded all taxa that were also highly prevalent in the healthy control samples while at the same time allowing to further analyze taxa that were enriched in matched normal samples such as blood as well as taxa that were dominant in tumor tissue. After this step, 147/218 (67.4%) potential tumor-linked species-level taxa remained. Next, taxa that were detected in fewer than 5 tumor tissue or matched normal samples were excluded (*n* = 78), as well as all remaining phages (*n* = 2). *Hepatitis B* as a known cancer-linked virus was re-included despite being detected in fewer than 5 tumor tissue or matched normal samples according to these criteria so that 68/218 (31.2%) taxa remained.

Taxa that survived this filtering strategy were likely to be tumor-linked but could also represent artifacts from contamination. To account for that, all taxa were filtered out that are known to be regularly present in the oral microbiome [69–71] and were at the same time mainly (> 50% of all RPPB matching a respective taxon) detected in samples that are likely contaminated with the oral microbiome (saliva matched normal, oral cancer tissue, and esophageal cancer tissue samples) (Supplementary data 8). For example, this filtered out taxa that are a commonly present in the oral human flora such as *Streptococcus mitis* and were indeed mainly detected in tumor tissue samples of oral cancer or esophageal cancer and likely a contaminant based on the biopsy location. Similarly, these taxa were detected in the matched normal of leukemia cases whose matched normal was a saliva sample likely containing taxa of the normal human microbiome. After this filtering step, 49/218 (22.5%) species-level taxa remained.

It has recently emerged that both reagents and kits used in DNA extraction and library preparation as well as ultrapure water used in laboratories can contain contaminants that can hamper the detection of truly present taxa in low biomass or high background (e.g., human) samples. As this study was performed using samples that were both low in non-human biomass and in the context of high human background, the aim was to further reduce false positives by compiling a list of common contaminants in microbiome studies. Recommended approaches, such as the sequencing of blank controls [72], were not feasible as the present study was conducted utilizing already sequenced primary material. Therefore, a database of common contaminants from various studies [73–77] examining this issue was compiled (Supplementary data 9). All previously recognized contaminant taxa apart from those that were described as a contaminant on the genus level but where different species within the genus were detected differently in 1000 genome control samples and matched normal or tumor tissue samples were excluded. This was the case for the genera *Pseudomonas* and *Methylobacterium*. While the species *Pseudomonas aeruginosa*, *Pseudomonas putida*, and *Pseudomonas stutzeri* were detected in both 1000 genome control and matched normal or tumor tissue samples and thus likely contaminants, *Pseudomonas fluorescens*, *Pseudomonas mendocina*, *Pseudomonas poae*, *Pseudomonas protegens*, and *Pseudomonas sp. TKP* were not detected in 1000 genome healthy control samples. Thus, it is likely that the species-level resolution of this analysis was able to differentiate between common contaminants and possibly tumor-linked taxa. Similarly, differentiation was possible between *Methylobacterium radiotolerans* and *Methylobacterium extorquens* (likely contaminants) and *Methylobacterium populi*, which was only found in tumor samples. The same held true for *Cupriavidus metallidurans* and *Propionibacterium propionicum*. Of note, the 1000 genome healthy control cohort used within this study contains samples processed and sequenced in 5 different sequencing centers (86 at the BGI-Shenzhen, 86 at the Broad Institute, 11 at Illumina, 113 at the Sanger Institute, and 69 at Washington University in St Louis). Thus, this control cohort itself serves as a bona fide contamination control including potential sequencing contaminants originating in different reagents, different suppliers, and different laboratory or environmental contaminants. After this filtering step, 27/218 (12.4%) species-level taxa remained (Fig. 1i,j, Supplementary Figure 6). While it cannot be ruled out that all these filtering steps removed truly cancer-linked taxa, the aim was to be cautious and rather accept a false-negative than a false positive finding. Of course, experimental validation of the relevance of one of the taxa eliminated by one of the filters could prove that this taxon is both, a sequencing contaminant and a relevant taxon in cancer.

If a taxon is indeed present in a tissue, matching read pairs are expected to be uniformly distributed across its genome. Consequently, a further filtering step was introduced. The sequencing data from all samples was combined and matched against a reference database constructed out of the genome of these 27 taxa. Next, the coverage distribution of reads across each taxon’s genome was assessed (Supplementary Figure 4). If detection of the respective taxa is the result from misalignment or sequence similarity between the taxa and for example cloning vectors used in the production of sequencing reagents, an uneven coverage would result. It was found that all read pairs matching *Human Mastadenovirus C* aligned to short parts of its genome with a maximum length of a few hundred base pairs and abrupt drops in coverage (Supplementary Figure 4). This was also found in another study using different cancer tissue sequencing data and resulted in excluding *Mastadenovirus C* from further analysis [4]. Using Blast (beginning with version 2.7.1 and subsequently updated) [78], it was found that read pairs matching *Human Mastadenovirus C* aligned all equally well to commonly used cloning vectors, such as pAxCALGL, which might have been used in the production of reagents used for sequencing. This form of contamination was recently analyzed and found to be frequent [79]. However, read pairs aligning to *Human Mastadenovirus C* originated from very few, seemingly unlinked samples from diverse cancer sequencing projects, making contamination by recombinant DNA unlikely. Another explanation for the observed coverage pattern is somatic genomic integration of parts of the *Human Mastadenovirus C* genome into a specific cancer genome. On balance, *Human Mastadenovirus C* was therefore not excluded from further analysis.

### Clustering of pipeline hits

t-SNE was performed using a web-based TensorFlow Embedding Projector implementation [80]. The learning rate and the perplexity were heuristically set to 10 and 30 for all analyses, respectively, except for the liver cancer subset, for which the perplexity was set to 50 due to improved cluster discrimination. The number of iterations was heuristically chosen, so that no major changes of cluster composition occurred upon increasing the number of iterations. Depending on the subset analysis, between 500 and 1500 iterations were needed to reach that point. t-SNE was performed in 3 dimensions for the pan-cancer analysis and in 2 dimensions for the cancer-specific analyses.

*k*-means clustering was performed using Morpheus [81]. Unsupervised *k*-means clustering using Euclidean distance as a similarity measure was employed with the number of clusters being heuristically informed by combining visual inspection, comparing t-SNE and *k*-means clusters and by examining marginal reduction of within-group variance with increasing numbers of clusters (i.e., the elbow method) (Supplementary Figure 9 A-J).

To combine the information obtained by both complimentary methods, t-SNE clustering was repeated with the same settings for each analysis, while color-coding clusters inferred from *k*-means clustering.

### Assessment of taxonomic differences between cell culture-derived and blood-derived DNA samples from the 1000 genome project

All taxa in the final taxon list (*n* = 218) (Supplementary data 10) which were identified in blood-derived 1000 genome healthy control samples were selected (*n* = 76) (Supplementary data 1). The pipeline was subsequently applied to randomly selected (identifier ending with 8 or 8) LCL-derived 1000 genome samples (*n* = 102) ( 2). Finally, RPPB in these LCL-derived samples were calculated for all 76 taxa that were identified in the blood-derived 1000 genome samples and compared between blood-derived and LCL-derived samples.

### Assessment of effect of subsampling of read pairs on relative taxon distribution

To show that subsampling alters neither the detected species-level taxa nor their relative composition compared to analyzing all non-human read pairs, a subset of 184 tumor tissue samples (Supplementary data 6) was analyzed, without any subsampling and subjected to the pipeline in the same way as in the main analysis. For example, read pairs matching *Human Mastadenovirus C*, *Pseudomonas poae*, *Ralstonia pickettii*, and *Propionibacterium acnes* were used to compare absolute read pair counts between full data and the subsample for all taxon-sample pairs in which the 10% subsample had at least 10 matches to the respective taxon.

### Concordance between WGS and RNA-seq

In order to assess the concordance between RNA-seq and WGS experiments performed on the same sample, the main pipeline was applied with the same settings to all samples with RNA-seq and WGS paired data available (*n* = 324). Pearson correlation coefficients of log_10_ transformed data were calculated for both a combined dataset of all RNA-seq / WGS pairs and for each sample for which RNA-seq and WGS data was available (*n* = 324).

### Assessment of somatic lateral gene transfer

In order to assess the integration of bacterial DNA into human DNA, read pairs with one read matching the human genome and one read matching one of the taxa in the final filtered taxon list (*n* = 27) (Supplementary data 10) were identified. First, representative bacterial and viral genomes for the final filtered taxon list were downloaded (Accession numbers in Supplementary data 10). These genomes were merged with the human reference genome (hg1k_v37, downloaded from the 1000 genome FTP server [59]) into one FASTA file using customized scripts. Second, all read pairs in which only one read of a read pair was mapped to the human genome were filtered using Samtools (version 1.7) [62]. All tumor tissue samples and matched normal samples of all patients in which at least 1000 RPPB matching one of the taxa in the final filtered taxon list were identified in that respective patient’s tumor tissue sample (*n* = 79, Supplementary data 12) and were included in this analysis. BWA mem (version 0.7.17) [82] with standard settings in paired end mode was used to align all such read pairs to the merged FASTA file of all taxa and the human reference genome. Next, all read pairs that were now divergently mapped were extracted using Samtools (version 1.7) [62] and customized scripts by only including read pairs where one read mapped to one of the included non-human taxa and the other read mapped to a human sequence. Only read pairs with a mapping quality of at least 40 were retained. Customized scripts were used to count and tabulate all obtained divergently mapped read pairs by taxa and sample, respectively (Supplementary data 13-14). In order to normalize read pairs mapping to putative integration sites (i.e., divergently mapped read pairs as defined above) by correcting for the total number of read pairs matching a taxon with a similar approach (i.e., non-divergently mapped, putatively non-integrated reads), a comparable pipeline was applied to the data used for the main analysis (i.e., both reads in a read pair not mapped to the human genome) of all patients included in the integration analysis (*n* = 79) (Supplementary data 12). First, the genomes of all taxa in the final filtered taxon list were downloaded (Accession numbers in Supplementary data 10) and merged without adding any further human sequences into one FASTA file to create a reference genome containing all taxa in the final filtered taxon list. BWA mem (version 0.7.17) [82] with standard settings in paired end mode was used to align these reads to the merged FASTA file of all taxa in the final filtered taxon list. Subsequently, Samtools (version 1.7) [62] was used to filter the aligned data to only include read pairs that mapped as a proper pair to only one taxon with a minimum mapping quality of 60. Samtools (version 1.7) [62] and customized scripts were used to count and tabulate all mapped reads. The integration rate of a taxon in either tumor tissue samples or matched normal samples was calculated by dividing the number of divergently mapping read pairs by the number of read pairs mapping as a proper pair to the respective taxon.

### Assessment of links of taxon-defined patient clusters to patient or cancer phenotypes

Differences in age at diagnosis between patient clusters were first analyzed by ANOVA for each cancer. All results with *p*_anova_ ≤ 0.1 are shown in Fig. 5. Such clusters or combinations of clusters were then compared to the other clusters by Student’s *t* test.

Differences in the gender distribution between patient clusters were analyzed by chi-square test for each cancer.

In order to analyze relationships between the number or type of cancer-associated somatic mutations and detection of specific taxa, all somatic mutations for all patients included in this study were obtained from the ICGC data portal [22, 58]. All synonymous mutations were filtered out. Subsequently, only Tier 1 cancer gene census [83] genes that were altered in more than 20 cases were filtered using customized scripts and included in the analysis (Supplementary data 23).

Differences in the number of somatic mutations in cancer genes between patient clusters were first analyzed by ANOVA for each cancer. All results with *p*_anova_ ≤ 0.1 are shown in Fig. 5. Such clusters or combinations of clusters were then compared to the other clusters by Student’s *t* test.

The Kaplan-Meier method was used to estimate survival curves for each patient cluster in each cancer type with available survival data. Differences in survival between patient clusters were analyzed by log rank test.

Links between patient clusters and somatic mutations in single cancer genes were analyzed if the gene was altered in at least 10 patients in that respective cancer type. Clusters or combinations of clusters were then compared to the other clusters by Fisher’s exact test.

All calculations were performed with R (beginning with version R 3.3.2. and subsequently updated) [68].

### Unbiased linkage analysis between single bacterial and viral taxa and patient or cancer phenotypes

For this analysis, a taxon was considered detected in a patient if at least 100 RPPB matched the respective taxa in the patient’s tumor tissue sample. All phages were excluded from the analysis. All included projects were grouped by cancer (Supplementary data 11). Cancer genes were defined as above.

All calculations were performed with R (beginning with version R 3.3.2. and subsequently updated) [68]. All *p* values were corrected for multiple testing using the FDR method to obtain a *q*-value, which was considered significant if < 0.05.

#### Survival analysis

First, the Kaplan-Meier method was used to estimate survival curves for each cancer-taxon pair that was detected in at least 10 patients. Differences in survival between patients with or without detection of a respective taxon were analyzed using log rank tests, stratified by ICGC project. Additionally, a pan-cancer analysis was performed in the same way, also stratifying by ICGC project.

#### Links between bacterial or viral taxa and patient gender

Links between detection of a taxa and patient gender were analyzed by Fisher’s exact test for each cancer-taxon pair that was detected in at least 10 patients. Additionally, a pan-cancer analysis was performed in the same way, using a logistic regression model that included the ICGC project as an independent variable.

#### Links between bacterial or viral taxa and patient age

Links between the detection of a taxon and patient age at diagnosis were analyzed by Student’s *t* test for each cancer-taxon pair that was detected in at least 10 patients. Additionally, a pan-cancer analysis was performed in the same way, using a linear regression model that was stratified by ICGC project.

#### Links between bacterial or viral taxa and number of somatic mutations in cancer genes

Links between the detection of a taxon and the number of non-synonymous somatic mutations in cancer consensus genes [83] of a patient were analyzed by Student’s *t* test for each cancer-taxon pair in which a taxon was detected in at least 10 patients. Additionally, a pan-cancer analysis was performed in the same way, using a linear regression model that was stratified by ICGC project.

#### Links between bacterial or viral taxa and specific somatic mutations

Links between the detection of a taxon and non-synonymous somatic mutations in one of the cancer consensus genes [83] of a patient were analyzed by Fisher exact test for each cancer-taxon pair that was detected in at least 10 patients. Additionally, a pan-cancer analysis was performed in the same way, using a logistic regression model that included the ICGC project as an independent variable.

### Assessment of differential gene expression

Reads per kilobase of transcript, per million mapped reads (RPKM) data was downloaded from http://dcc.icgc.org/pcawg for all available tumor tissue samples. Ensemble gene ID was substituted by the standard Human Genome Organization (HUGO) Gene Nomenclature Committee (HGNC) symbol downloaded from http://genenames.org/download/custom and the RPKM data was then linked to the identified species-level taxa in each sample using R (beginning with version R 3.3.2. and subsequently updated) [68]. sRAP [84] was used to normalize RPKM values, perform quality control and differential gene expression analysis, and to identify pathways that are differentially expressed. For these analyses, each cancer was analyzed separately and patients that had more than 100 RPPB matching one species-level taxon in the final filtered taxon list were compared with those who did not. Differential gene expression analysis was performed for all taxa that were detected with more than 100 RPPB in at least five patients and RNA-seq data available (Supplementary data 15). Next, sRAP [84] was used to perform bidirectional functional enrichment of gene expression data to identify pathways up- or downregulated between patients with or without detection of a taxon. Briefly, the distribution of activation versus inhibition *t*-test statistics for all samples linked or not linked to a specific taxon was compared using ANOVA and corrected for multiple testing using the FDR method [84]. A gene set was considered functionally enriched if *q* < 0.05. Gene sets likely not relevant for the respective cancer were excluded and gene sets provided with sRAP [84] were reduced to gene ontology (GO) gene sets [85].

## Supplementary Information

**Additional file 1: Supplementary Table 1.** Included patients and samples. **Supplementary Figure 2.** Validation of pipeline and analytical approach. A, mean RPPB detected for indicated species in blood-derived and lymphoblastoid cell line 1000 Genome samples sorted by mean of blood-derived samples. B, proportion of non-human read pairs matching the indicated taxon of read pairs matching any species-level taxon for each external validation sample. C, comparison of Kraken matched read pairs in RNA-seq and WGS data of the same sample. Each dot represents one species-level taxon in one sample with both RNA-seq and WGS data available. The line represents the best-fitted line (log-log linear regression). Pearson correlation coefficients (log-log) are shown with two-sided p-values. D, plot of Pearson correlation coefficient (log-log) distribution of all samples with both RNA-seq and WGS data available. Each dot represents the Pearson correlation coefficient within a single sample. E, comparison of 10% subsample and full dataset. Each dot represents one species-level taxon in one sample for which both the full and the subsampled dataset has been analyzed and indicates the absolute read count identified in both samples. The line represents the best-fitted line (log-log linear regression). Pearson correlation coefficients (log-log) are shown with two-sided p-values. F, Ratio of absolute read counts in the full sample to the 10% subsample for 4 selected taxa. The mean ratio of all samples in which the respective taxon was detected is indicated by the symbol and the error bars indicate the standard error of the mean. The dotted line shows the expected ratio of 10. G, comparison of tumor tissue and matched normal by patient and taxon. Each dot represents one species-level taxon in one patient with both tumor tissue and matched normal analyzed and indicates the RPPB in both samples. The line represents the best-fitted line (log-log linear regression). Pearson correlation coefficients (log-log) are shown with two-sided p-values. **Supplementary Figure 3.** Alpha diversity. A, counts of 1000 Genome samples by species-level richness. B, counts of tumor tissue samples by species-level richness color-coded by project. C, counts of matched normal samples by species-level richness color-coded by project. D, comparison of richness between projects and sample type. Bars show mean and error bars standard deviation. N in brackets indicates total sample number for each project. **Supplementary Figure 4.** Coverage distribution for all tumor-linked species-level taxa. Coverage distribution across each species-level taxon identified as tumor-linked. **Supplementary Figure 5.** Flow chart of taxa filtering strategy. Flow chart of filtering strategy to derive likely tumor-linked species-level taxa. **Supplementary Figure 6.** Heatmap of filtered taxons. Log_2_-transformed RPPB of all species-level taxa identified as likely tumor-linked after filtering in all samples. Taxa were hierarchically clustered using Pearson correlation as a distance measure with average-linkage. Samples were hierarchically clustered within each project and type subgroup using Pearson correlation as a distance measure with average-linkage. **Supplementary Figure 7.** Heatmaps of tumor-linked taxa for all cancers without discernible clusters. A-D, log_2_-transformed RPPB of all species-level taxa identified as tumor-linked and detected after filtering in all tumor-tissues of the indicated cancer. Results of *k*-means clustering of samples are shown. **Supplementary Figure 8.** Host integration. A, integration rate by species for tumor tissue and matched normal sample. B, difference in integration rates between bacterial and viral taxa (p < 0.0001, Wilcoxon rank-sum test, two-tailed). The midline of the boxplot shows the median, the box borders show upper and lower quartile, the whiskers show 5^th^ and 95^th^ percentiles and the dots outliers of species-specific integration rates in tumor tissue or matched normal samples. C, difference in integration rate between tumor tissue and matched normal samples (*p* = 0.0009, Wilcoxon signed rank test, two-tailed). The midline of the boxplot shows the median, the box borders show upper and lower quartile, the whiskers show 5^th^ and 95^th^ percentiles and the dots outliers of species-specific integration rates. **Supplementary Figure 9.** “Elbow method” to determine k for k-means clustering. A-J, plot of reducing within group sum of squares for increasing k (number of clusters) in k-means clustering (Supplementary Figures 4 and 5) of log_2_-transformed RPPB of all species-level taxa identified as tumor-linked and detected after filtering in all tumor-tissues for each indicated cancer.

**Additional file 2: Supplement_1-3.** control and validation. **Supplement_4.** rppb and read counts raw data_revised. **Supplement_5.** downsampling vs full dataset. **Supplement_6.** number of species detected in each sample. **Supplement_7-9.** filtering and grouping. **Supplement_10.** final taxa hit list. **Supplement_11.** cancer study groupings. **Supplement_12-14.** integration analysis. **Supplement_15.** differential gene expression analysis overview. **Supplement_16-22.** differential gene expression and pathway analysis. **Supplement_23.** most commonly mutated genes in dataset.

**Additional file 3:.** Supplementary note.

## Data Availability

All raw data generated in this study is included in the supplement. Original sequencing datasets are available here: https://dcc.icgc.org/. To access original sequencing data, a request has been made to the ICGC DACO, which will be granted according to the conditions set out here: https://icgc.org/index.php?q=daco.
