# Supplementary material for: An atlas of the tissue and blood metagenome in cancer reveals novel links between bacteria, viruses and cancer
Source: Microbiome. 2021 Apr 22;9:94. doi: 10.1186/s40168-021-01039-4 (PMC8063312; doi:10.1186/s40168-021-01039-4)
Supplement: Supplementary file 2 — Additional file 1: Supplementary Table 1. Included patients and samples. Supplementary Figure 2. Validation of pipeline and analytical approach. A, mean RPPB detected for indicated species in blood-derived and lymphoblastoid cell line 1000 Genome samples sorted by mean of blood-derived samples. B, proportion of non-human read pairs matching the indicated taxon of read pairs matching any species-level taxon for each external validation sample. C, comparison of Kraken matched read pairs in RNA-seq and WGS data of the same sample. Each dot represents one species-level taxon in one sample with both RNA-seq and WGS data available. The line represents the best-fitted line (log-log linear regression). Pearson correlation coefficients (log-log) are shown with two-sided p-values. D, plot of Pearson correlation coefficient (log-log) distribution of all samples with both RNA-seq and WGS data available. Each dot represents the Pearson correlation coefficient within a single sample. E, comparison of 10% subsample and full dataset. Each dot represents one species-level taxon in one sample for which both the full and the subsampled dataset has been analyzed and indicates the absolute read count identified in both samples. The line represents the best-fitted line (log-log linear regression). Pearson correlation coefficients (log-log) are shown with two-sided p-values. F, Ratio of absolute read counts in the full sample to the 10% subsample for 4 selected taxa. The mean ratio of all samples in which the respective taxon was detected is indicated by the symbol and the error bars indicate the standard error of the mean. The dotted line shows the expected ratio of 10. G, comparison of tumor tissue and matched normal by patient and taxon. Each dot represents one species-level taxon in one patient with both tumor tissue and matched normal analyzed and indicates the RPPB in both samples. The line represents the best-fitted line (log-log linear regression). Pearson correlation coefficients [file 40168_2021_1039_MOESM2_ESM.docx]

SUPPLEMENTARY FIGURES & TABLES

**Supplementary Table 1: Included patients and samples**

**Supplementary Figure 2: Validation of pipeline and analytical approach**

**Supplementary Figure Legend 2**

A, mean RPPB detected for indicated species in blood derived and lymphoblastoid cell line 1000 Genome samples sorted by mean of blood derived samples. B, proportion of non-human read pairs matching the indicated taxon of read pairs matching any species-level taxon for each external validation sample. C, comparison of Kraken matched read pairs in RNA-seq and WGS data of the same sample. Each dot represents one species-level taxon in one sample with both RNA-seq and WGS data available. The line represents the best-fitted line (log-log linear regression). Pearson correlation coefficients (log-log) are shown with two-sided p-values. D, plot of Pearson correlation coefficient (log-log) distribution of all samples with both RNA-seq and WGS data available. Each dot represents the Pearson correlation coefficient within a single sample. E, comparison of 10% subsample and full dataset. Each dot represents one species-level taxon in one sample for which both the full and the subsampled dataset has been analyzed and indicates the absolute read count identified in both samples. The line represents the best-fitted line (log-log linear regression). Pearson correlation coefficients (log-log) are shown with two-sided p-values. F, Ratio of absolute read counts in the full sample to the 10% subsample for 4 selected taxa. The mean ratio of all samples in which the respective taxon was detected is indicated by the symbol and the error bars indicate the standard error of the mean. The dotted line shows the expected ratio of 10. G, comparison of tumor tissue and matched normal by patient and taxon. Each dot represents one species-level taxon in one patient with both tumor tissue and matched normal analyzed and indicates the RPPB in both samples. The line represents the best-fitted line (log-log linear regression). Pearson correlation coefficients (log-log) are shown with two-sided p-values.

**Supplementary Figure 3: Alpha diversity**

**Supplementary Figure Legend 3**

A, counts of 1000 Genome samples by species-level richness. B, counts of tumor tissue samples by species-level richness color-coded by project. C, counts of matched normal samples by species-level richness color-coded by project. D, comparison of richness between projects and sample type. Bars show mean and error bars standard deviation. N in brackets indicates total sample number for each project.

**Supplementary Figure 4: Coverage distribution for all tumor-linked species-level taxa**

**Supplementary Figure Legend 4**

Coverage distribution across each species-level taxon identified as tumor-linked.

**Supplementary Figure 5: Flow chart of taxa filtering strategy**

**Supplementary Figure Legend 5**

Flow chart of filtering strategy to derive likely tumor-linked species-level taxa.

**Supplementary Figure 6: Heatmap of filtered taxons**


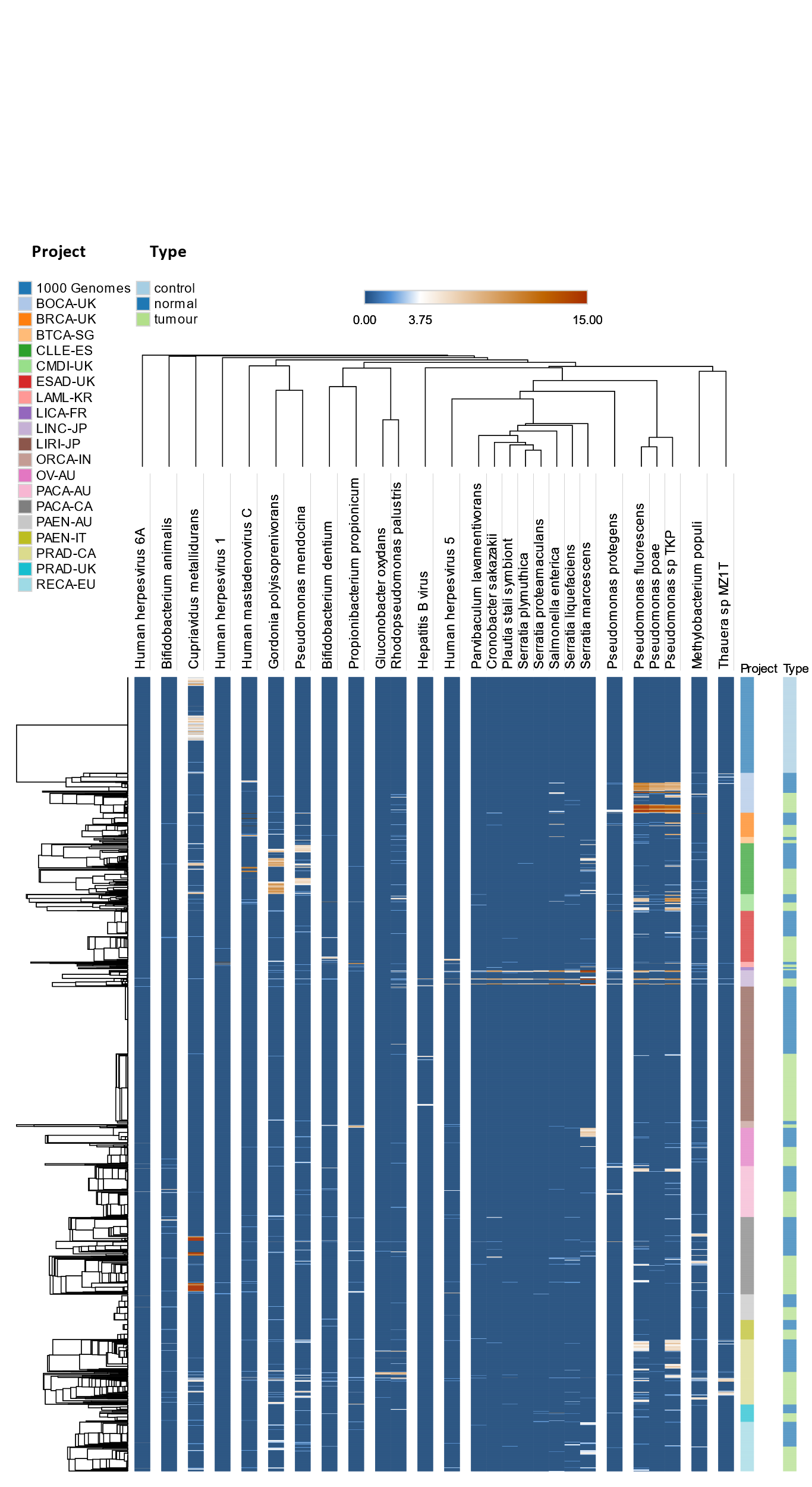


**Supplementary Figure Legend 6**

Log_2_-transformed RPPB of all species-level taxa identified as likely tumor-linked after filtering in all samples. Taxa were hierarchically clustered using Pearson correlation as a distance measure with average-linkage. Samples were hierarchically clustered within each project and type subgroup using Pearson correlation as a distance measure with average-linkage.

**Supplementary Figure 7: Heatmaps of tumor-linked taxa for all cancers without discernible clusters**


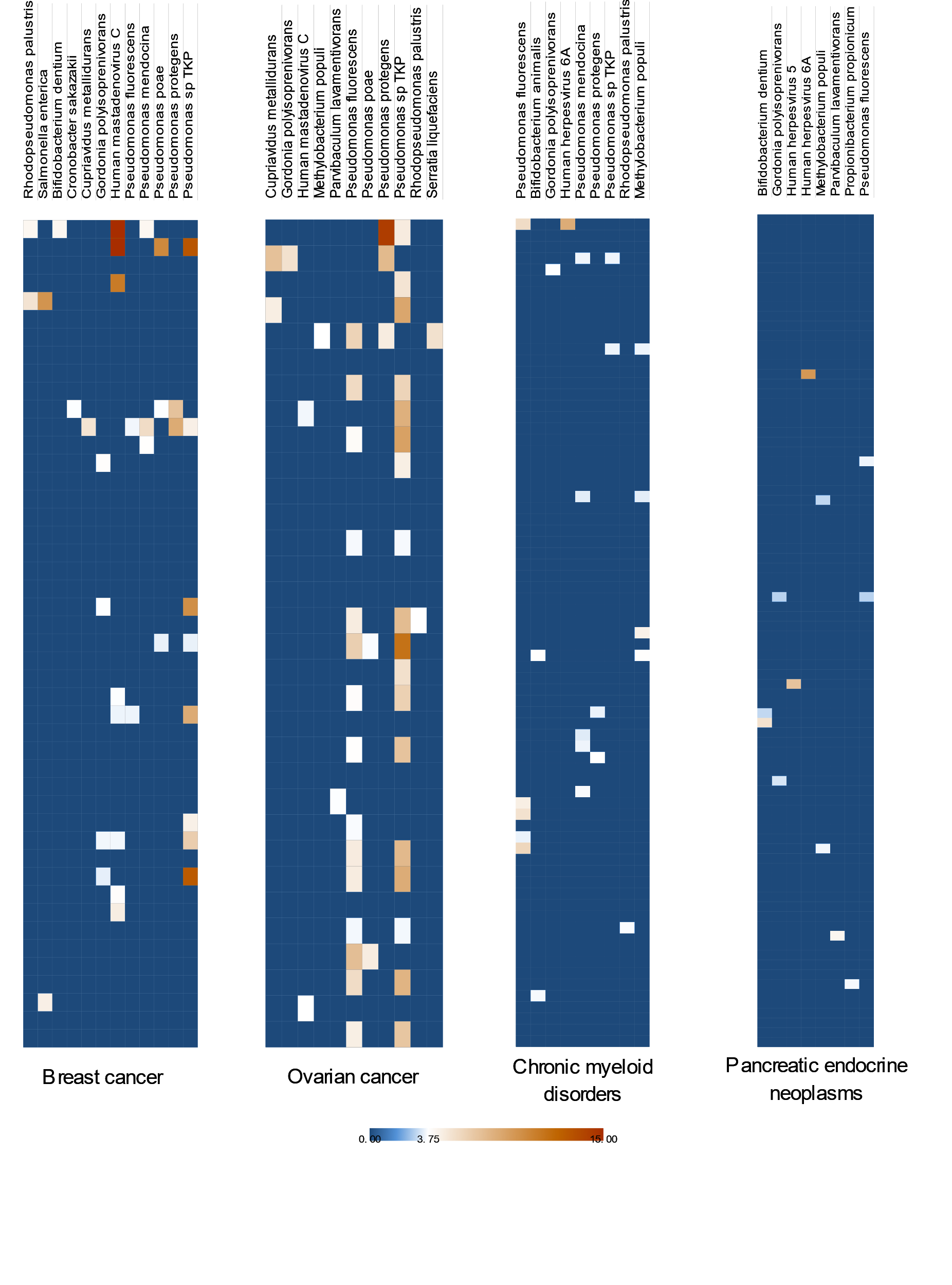


**Supplementary Figure Legend 7**

A-D, log_2_-transformed RPPB of all species-level taxa identified as tumor-linked and detected after filtering in all tumor-tissues of the indicated cancer. Results of K-means clustering of samples are shown.

**Supplementary Figure 8: Host integration**

**Supplementary Figure Legend 8**

A, integration rate by species for tumor tissue and matched normal sample. B, difference in integration rates between bacterial and viral taxa (p<0.0001, Wilcoxon rank-sum test, two-tailed). The midline of the boxplot shows the median, the box borders show upper and lower quartile, the whiskers show 5^th^ and 95^th^ percentiles and the dots outliers of species-specific integration rates in tumor tissue or matched normal samples. C, difference in integration rate between tumor tissue and matched normal samples (p=0.0009, Wilcoxon signed-rank test, two-tailed). The midline of the boxplot shows the median, the box borders show upper and lower quartile, the whiskers show 5^th^ and 95^th^ percentiles and the dots outliers of species-specific integration rates.

**Supplementary Figure 9: „Elbow method“ to determine k for k-means clustering**

**Supplementary Figure Legend 9**

A-J, plot of reducing within group sum of squares for increasing k (number of clusters) in k-means clustering (Supplementary figure 4, Supplementary figure 5) of log_2_-transformed RPPB of all species-level taxa identified as tumor-linked and detected after filtering in all tumor-tissues for each indicated cancer.
