# Supplementary material for: An atlas of the tissue and blood metagenome in cancer reveals novel links between bacteria, viruses and cancer
Source: Microbiome. 2021 Apr 22;9:94. doi: 10.1186/s40168-021-01039-4 (PMC8063312; doi:10.1186/s40168-021-01039-4)
Supplement: Supplementary file 4 — Additional file 3:. Supplementary note. [file 40168_2021_1039_MOESM4_ESM.docx]

Supplementary note

**An atlas of the tissue and blood metagenome in cancer reveals novel links between bacteria, viruses and cancer**

**Validation of Pipeline**

It was confirmed that the pipeline was able to identify already known bacterial and viral taxa in tissue-derived bacterial isolates and cell lines with known integration of viral DNA. First, the pipeline was applied to a gastric mucosa isolate of Helicobacter pylori from a stomach ulcer patient (sample ID: SRS2800545, run ID: SRR6432709). As expected, 3,606,537/3,607,687 (99.97%) of read pairs matching any taxon in the database matched Helicobacter pylori (Supplementary Data 3, Supplementary Figure 2B). Second, the pipeline was applied to 3 cell line samples: HeLa (run ID: SRR1611000), CaSki (run ID: SRR1611128) and SiHa (run ID: SRR1611127). These 3 cell lines contain known integrations of Human papillomavirus in their genome^26^. Applying the pipeline resulted in between 78.14% and 99,90% of non-human read pairs matching any species-level taxon in the database to match Human papillomavirus 7 or 9 (Supplementary Data 3, Supplementary Figure 2B). Interestingly, the majority of non-human read pairs not matching Human papillomavirus 7 or 9 matched Mycoplasma hyorhinis (0.08%-21.74%), a common cell culture contaminant^27^. Thus, it was confirmed that the pipeline can identify bacterial and viral taxa irrespective of both a high level of human background DNA and whether the identifying sequence is present in the form of host-integrated DNA or by the presence of the viral or bacterial taxa itself in the sample.

Finally, the pipeline was independently run with the same settings on available matched RNA-Seq and WGS data of the same tumor tissue samples. It was shown that identified taxa in pairs of RNA-Seq and WGS data of the same tumor tissue sample are correlated both in a combined dataset of all pairs and within each sample for which RNA-Seq and WGS data was available (n=324) (Supplementary Figure 2 C-D). This validation is especially useful to minimize the risk of contamination post sample processing.
